# Comprehensive behavioral analysis of voltage-gated calcium channel beta-anchoring and -regulatory protein knockout mice

**DOI:** 10.3389/fnbeh.2015.00141

**Published:** 2015-06-16

**Authors:** Akito Nakao, Takafumi Miki, Hirotaka Shoji, Miyuki Nishi, Hiroshi Takeshima, Tsuyoshi Miyakawa, Yasuo Mori

**Affiliations:** ^1^Division of Systems Medical Science, Institute for Comprehensive Medical Science, Fujita Health UniversityToyoake, Japan; ^2^Department of Synthetic Chemistry and Biological Chemistry, Graduate School of Engineering, Kyoto UniversityKyoto, Japan; ^3^Japan Science and Technology Agency, Core Research for Evolutional Science and TechnologyKawaguchi, Japan; ^4^Department of Biological Chemistry, Graduate School of Pharmaceutical Sciences, Kyoto UniversityKyoto, Japan; ^5^Section of Behavior Patterns, Center for Genetic Analysis of Behavior, National Institute for Physiological SciencesOkazaki, Japan

**Keywords:** voltage-gated calcium channels, voltage-gated calcium channel beta-anchoring and -regulatory protein, knockout mouse, behavior, psychiatric disorders

## Abstract

Calcium (Ca^2+^) influx through voltage-gated Ca^2+^ channels (VGCCs) induces numerous intracellular events such as neuronal excitability, neurotransmitter release, synaptic plasticity, and gene regulation. It has been shown that genes related to Ca^2+^ signaling, such as the *CACNA1C*, *CACNB2*, and *CACNA1I* genes that encode VGCC subunits, are associated with schizophrenia and other psychiatric disorders. Recently, VGCC beta-anchoring and -regulatory protein (BARP) was identified as a novel regulator of VGCC activity via the interaction of VGCC β subunits. To examine the role of the BARP in higher brain functions, we generated BARP knockout (KO) mice and conducted a comprehensive battery of behavioral tests. BARP KO mice exhibited greatly reduced locomotor activity, as evidenced by decreased vertical activity, stereotypic counts in the open field test, and activity level in the home cage, and longer latency to complete a session in spontaneous T-maze alteration test, which reached “study-wide significance.” Acoustic startle response was also reduced in the mutants. Interestingly, they showed multiple behavioral phenotypes that are seemingly opposite to those seen in the mouse models of schizophrenia and its related disorders, including increased working memory, flexibility, prepulse inhibition, and social interaction, and decreased locomotor activity, though many of these phenotypes are statistically weak and require further replications. These results demonstrate that BARP is involved in the regulation of locomotor activity and, possibly, emotionality. The possibility was also suggested that BARP KO mice may serve as a unique tool for investigating the pathogenesis/pathophysiology of schizophrenia and related disorders. Further evaluation of the molecular and physiological phenotypes of the mutant mice would provide new insights into the role of BARP in higher brain functions.

## Introduction

In calcium (Ca^2+^) signaling pathways, voltage-gated calcium channels (VGCCs) initiate numerous intracellular events including neuronal excitability, neurotransmitter release, synaptic plasticity, and Ca^2+^-induced gene regulation (Catterall, 2011). VGCCs are heteromultimeric protein complexes composed of the pore-forming α1 subunit, designated as CaV, and auxiliary subunits α2/δ, β, and γ (Ertel et al., 2000). The α1 and β subunits interact to enhance functional channel trafficking to the plasma membrane (Mori et al., 1991; Bichet et al., 2000), to modify multiple kinetic properties (Varadi et al., 1991), and to regulate the stability of VGCC complexes (Campiglio et al., 2013). β subunits are thought to be the structural and functional platform of VGCC complexes, because they contain an SH3-HOOK-GK module that places them within a family of proteins called the membrane-associated guanylate kinases (Buraei and Yang, 2010; Nakao et al., 2013). The β subunits directly interact with various proteins, including small G-proteins such as Kir/Gem, Rem, and Rad, to regulate VGCC activity (Béguin et al., 2001; Finlin et al., 2003). At the presynaptic active zones, β subunits interact with Rab3-interacting molecules (RIMs), Bassoon, and CAST (Kiyonaka et al., 2007, 2012; Uriu et al., 2010; Chen et al., 2011), and these interactions are critical for VGCCs and active zone proteins to couple functionally to regulate neurotransmitter release. Interestingly, several studies have demonstrated that the β4 subunit directly regulates activity-dependent gene regulation (Subramanyam et al., 2009; Tadmouri et al., 2012). In this context, the β4 subunit also binds to a transcription factor for DNA binding, a phosphatase for histone dephosphorylation, and heterochromatin protein 1γ for nucleosome association (Hibino et al., 2003; Xu et al., 2011; Tadmouri et al., 2012). Recently, VGCC beta-anchoring and -regulatory protein (BARP) was identified (Béguin et al., 2014). BARP modulates the localization of β subunits and their association with the α1 subunit to negatively regulate VGCC activity (Béguin et al., 2014).

Among the genes involved in psychiatric disorders, genes encoding VGCC subunits have attracted attention (Ripke et al., 2014). In 2002, a family-based association study revealed for the first time that CACNA1C, which encodes the VGCC pore-forming α1 subunit, is associated with bipolar disorder (Sklar et al., 2002). Genome-wide association studies have identified that CACNA1C and CACNB3, which encodes the VGCC β subunit, are involved in the development of bipolar disorder (Ferreira et al., 2008; Ripke et al., 2011; Sklar et al., 2011). Notably, the latest large-scale genome-wide association study shows that CACNA1C is ranked fourth in terms of the degree of association (*p*-value) with schizophrenia out of 108 schizophrenia-associated genetic loci (Ripke et al., 2014). In addition, CACNB2 and CACNA1I are nineteenth and thirty-ninth, respectively, of the 108 loci (Ripke et al., 2014).

CaV1.2, encoded by CACNA1C, has been shown to play critical roles in higher brain function in mouse models. Conditional deletion of CACNA1C in the mouse hippocampus and cortex resulted in a severe impairment of hippocampus-dependent spatial memory in the Morris water maze test (Moosmang et al., 2005; White et al., 2008). Acute pharmacological blockade of CaV1.2, but not chronic genetic inactivation, impaired acquisition of fear learning (Langwieser et al., 2010). Furthermore, an anterior cingulate cortex-limited deletion of CACNA1C in mice impaired observational fear learning (Jeon et al., 2010). Constitutive CACNA1C heterozygous mice, forebrain-specific conditional CACNA1C knockout (KO) mice, and prefrontal cortex-specific CACNA1C knockdown mice show increased anxiety-like behavior in the elevated plus maze (Lee et al., 2012).

It has been reported that genes encoding VGCC subunits, especially CACNA1C, are associated with psychiatric disorders (Sklar et al., 2002, 2011; Ferreira et al., 2008; Ripke et al., 2011, 2014). Considering the fact that BARP regulates VGCC activity including CaV1.2 encoded by CACNA1C (Béguin et al., 2014), we hypothesized that BARP deficiency may result in some behavioral abnormalities related to psychiatric disorders. We generated BARP KO mice using gene targeting by homologous recombination, and then conducted a comprehensive behavioral test battery, which covers many distinct behavioral domains from simple sensorimotor functions to higher brain functions including learning and memory, on the BARP KO mice (Powell and Miyakawa, 2006; Takao and Miyakawa, 2006a).

## Materials and methods

### Generation of mutant mice

The targeting vector was constructed using the genomic PCR products from 129/SvJ mouse genome DNA, synthetic linkers carrying the loxP sequence (Shibata et al., 1997), the neomycin-resistance gene from pMC1 Neo (Stratagene), the diphtheria toxin gene from pMC1-DT-A, and pBluscriptII SK(−) (Stratagene). J1 ES cells were transfected with the linearized targeting vector and selected using G418. Of ~550 clones isolated, Southern blotting analysis identified the clone carrying the homologous mutation. Chimeric mice produced with the ES clones were crossed with C57BL/6J mice and could transmit the mutant gene to the pups. To determine the mouse genotype, PCR with the primer sets of mDos_genoR_Not(+), mDos_genoL_Not2(−), and LacZ_Aat(−) was carried out using genomic DNA from mice; sequences are mDos_genoRNot(+) (5′-GAGCTGAACCTGAGCTGGCTCTATG-3′), mDos_genoL_Not2(−) (5′-GTGGGTGCCGTTGTCCAGATAAGTAG-3′), and LacZ_Aat(−) (5′-CGCTGATTTGTGTAGTCGGTTTATG-3′). For β-galactosidase staining, mice were perfused with a 2% paraformaldehyde solution buffered with 0.12 M sodium phosphate (pH 7.4) under pentobarbital anesthesia, and the brains were immersed immediately in ice-cold 25% sucrose. Frozen coronal sections (40 μm thickness) were stained with the X-Gal reagent (Nishi et al., 1997).

### Animals and experimental design

We used male BARP KO mice (*n* = 22), which were backcrossed to the C57BL/6J background for at least seven generations, and their male wild type (WT) littermates (*n* = 22). The ages of the mice at the time of the experiment are shown in Supplementary Table 1. Wild-type and mutant mice were group housed (total three to five per cage; one to three for each genotype) in a room with a 12 h light/dark cycle (lights on at 7:00 a.m.), with access to food and water *ad libitum*. Room temperature was kept at 23 ± 2°C. Behavioral testing was performed between 9:00 a.m. and 6:00 p.m. After the tests, all apparatus was cleaned with diluted sodium hypochlorite solution to prevent a bias due to olfactory cues. To minimize the effects of previous test experiment on subsequent behavior, we performed the behavioral test battery in a specific order, in which the less stressful tests preceded the more stressful tests (Supplementary Table 1). Each behavioral test was separated from the others at least by 1 day. All behavioral testing procedures were approved by the Institutional Animal Care and Use Committee of Fujita Health University.

### Behavioral tests

Most of the behavioral tests were performed as previously described (Yamasaki et al., 2008), unless otherwise noted.

#### Neurological screen and neuromuscular strength test

The righting, whisker twitch, and ear twitch reflexes were evaluated. Physical features, including the presence of whiskers or bald hair patches, were also recorded. A grip strength meter (O'HARA & Co., Tokyo, Japan) was used to assess forelimb grip strength. Mice were lifted by holding the tail so that their forepaws could grasp a wire grid. The mice were then gently pulled backward by the tail with their posture parallel to the surface of the table until they released the grid. The peak force applied by the forelimbs of the mouse was recorded in Newtons (N). Each mouse was tested three times, and the greatest value measured was used for data analysis. In the wire hang test, the mouse was placed on a wire mesh that was then slowly inverted, so that the mouse gripped the wire in order not to fall off. Latency to fall was recorded, with a 60 s cut-off time.

#### Light/dark transition test

A light/dark transition test was conducted as previously described (Takao and Miyakawa, 2006b). The apparatus consisted of a cage (21 × 42 × 25 cm) divided into two sections of equal size by a partition with a door (O'HARA & Co., Tokyo, Japan). One chamber was brightly illuminated (390 lx), whereas the other chamber was dark (2 lx). Mice were placed into the dark chamber and allowed to move freely between the two chambers with the door open for 10 min. The total number of transitions, latency to first enter the lit chamber, distance traveled, and time spent in each chamber were recorded by ImageLD software (see Section, “Data Analysis”).

#### Open field test

Each mouse was placed in the corner of the open field apparatus (40 × 40 × 30 cm; Accuscan Instruments, Columbus, OH, USA). The apparatus was illuminated at 100 lx. Total distance traveled (cm), vertical activity (rearing measured by counting the number of photobeam interruptions), time spent in the center area (20 × 20 cm), and beam-break counts for stereotyped behaviors (defined by the number of breaks of the same beam) were recorded. Data were collected for 120 min.

#### Elevated plus maze test

An elevated plus maze test was conducted as previously described (Komada et al., 2008). The elevated plus maze consisted of two open arms (25 × 5 cm) and two enclosed arms of the same size with 15 cm high transparent walls, and the arms were connected by a central square (5 × 5 cm) (O'HARA & Co., Tokyo, Japan). The open arms were surrounded by a raised ledge (3 mm thick and 3 mm high) to avoid mice falling off the arms. The arms and central square were made of white plastic plates and were elevated 55 cm above the floor. Arms of the same type were located opposite from each other. Each mouse was placed in the central square of the maze, facing one of the enclosed arms. The number of entries into the open and enclosed arms and the time spent in the open or enclosed arms were recorded during a 10-min test period. Percentage of entries into open arms, time spent in open arms (s), number of total entries, and total distance traveled (cm) were calculated. Data acquisition and analysis were performed automatically, using ImageEP software (see Section, “Data analysis”).

#### Hot plate test

The hot plate test was used to evaluate sensitivity to a painful stimulus. Mice were placed on a 55.0°C (± 0.1°C) hot plate (Columbus Instruments, OH, USA), and latency to the first fore- or hind-paw response was recorded. The paw response was defined as either a paw lick or a foot shake.

#### Social interaction test in a novel environment

In the social interaction test, two mice of identical genotypes that were previously housed in different cages were placed in a box together (40 × 40 × 30 cm) (O'HARA & Co., Tokyo, Japan) and allowed to explore freely for 10 min. Behavior was recorded and analyzed automatically using ImageSI program (see Section, “Data analysis”). The total number of contacts, total duration of active contacts, total contact duration, mean duration per contact, and total distance traveled were measured. If the two mice contacted each other and the distance traveled by either mouse was longer than 10 cm, the behavior was classified as an “active contact.” Images were captured at 3 frame per s, and distance traveled between two successive frames was calculated for each mouse.

#### Rotarod test

Motor coordination and balance were tested with the rotarod test. This test, which uses an accelerating rotarod (UGO Basile, Comerio, VA, Italy), was performed by placing mice on rotating drums (3 cm diameter) and measuring the time each animal was able to maintain its balance on the rod. The speed of the rotarod accelerated from 4 to 40 rpm over a 5-min period.

#### Crawley's sociability and social novelty preference test

This test is a well-designed method to investigate the effect of complex genetics on sociability and preference for social novelty (Crawley, 2004; Moy et al., 2004). The testing apparatus consisted of a rectangular, three-chambered box and a lid with an infrared video camera (O'HARA & Co., Tokyo, Japan). Each chamber was 20 × 40 × 47 cm and the dividing walls were made from clear Plexiglas, with small square openings (5 × 3 cm) allowing access into each chamber. We modified the method described by Moy et al. (2004) as follows: a habituation session was performed in the apparatus for 10 min the day before the sociability test, and the wire cages in the lateral compartments were located in the corners of each compartment. In the sociability test, an unfamiliar C57BL/6J male mouse (stranger), that had no prior contact with the subject mice, was placed in one of the side chambers. The location of the stranger mouse (stranger side) in the left vs. right side chamber was systematically alternated between trials. The stranger mouse was enclosed in a small round wire cage, which allowed nose contact between the bars, but prevented fighting. The cage was 11 cm in height, with a bottom diameter of 9 cm, and vertical bars 0.5 cm apart. The subject mouse was first placed in the middle chamber and allowed to explore the entire test box for a 10-min session. The amount of time spent in each chamber was measured with a camera fitted on top of the box. In the social novelty preference test, each mouse was tested in a 10-min session to quantify social preference for a new stranger. After the first 10-min session, a second unfamiliar mouse was placed in the chamber that had been empty during the first 10-min session. This second stranger was also enclosed in an identical small wire cage. The test mouse thus had a choice between the first, already-investigated unfamiliar mouse (familiar side), and the novel unfamiliar mouse (unfamiliar side). The amount of time spent in each chamber during the second 10-min session was measured as described above. Data acquisition and analysis were performed automatically using ImageCSI (see Section, “Data analysis”).

#### Startle response/prepulse inhibition (PPI) test

A startle reflex measurement system (O'HARA & Co., Tokyo, Japan) was used to measure acoustic startle response and PPI. A test session began by placing a mouse in a plastic cylinder where it was left undisturbed for 10 min. White noise (40 ms) was used as the startle stimulus for all trial types. The startle response was recorded for 400 ms (measuring the response every 1 ms) starting with the onset of the startle stimulus. The background noise level in each chamber was 70 dB. The peak startle amplitude recorded during the 140 ms sampling window was used as the dependent variable. A test session consisted of six trial types (i.e., 2 types for startle stimulus-only trials, and 4 types for PPI trials). The intensity of the startle stimulus was 110 or 120 dB. The prepulse sound was presented 100 ms before the startle stimulus, and its intensity was 74 or 78 dB. Four combinations of prepulse and startle stimuli were used (74–110, 78–110, 74–120, and 78–120 dB). Six blocks of the six trial types were presented in a pseudo-random order, such that each trial type was presented once within a block. The average inter-trial interval was 15 s (range 10–20 s).

#### Porsolt forced swim test

A transparent plastic cylinder (20 cm height × 10 cm diameter) filled with water (21–23°C) up to a height of 7.5 cm was put in a white plastic chamber (31 × 41 × 41 cm) (O'HARA & Co., Tokyo, Japan). Mice were placed into the cylinder, and both immobility and the distance traveled were recorded over a 10-min test period. Images were captured at 2 frame per s. For each pair of successive frames, the amount of area (pixels) within which the mouse moved was measured. When the amount of area was below a certain threshold, mouse behavior was classified as “immobile.” When the amount of area equaled or exceeded the threshold, the mouse was classified as “moving.” The optimal threshold to judge moving was determined by adjusting it to the amount of immobility measured by human observation. Immobility lasting for less than 2 s was not included in the analysis. Data acquisition and analysis were performed automatically, using ImageTS/PS software (see Section, “Data analysis”).

#### Barnes maze test

The Barnes maze task was conducted on “dry land,” a white circular surface 1.0 m in diameter, with 12 holes equally spaced around the perimeter (O'HARA & Co., Tokyo, Japan). A black Plexiglas escape box (17 × 13 × 7 cm), which had paper cage bedding on its bottom, was located under one of the holes. The hole above the escape box represented the target, analogous to the hidden platform in the Morris water maze task. The location of the escape box (target) was consistent for a given mouse but randomized across mice. The maze was rotated daily, with the spatial location of the target unchanged with respect to the distal visual room cues, to prevent a bias based on olfactory or proximal cues within the maze. The mice that could not find the box were guided to it and allowed to enter it to remain there for 30 s. Two trials per day were conducted for 6 successive days. Each trial ended when the mouse entered the escape box or after 5 min had elapsed. The amount of time that the mice took to enter the box (escape latency), the number of errors (defined by the animal placing its nose in a hole that did not lead to the escape box), and the number of omission errors (defined by the visit to the target hole without subsequent entry into the target hole) were recorded by ImageBM software. On day 7, a probe test was conducted without the escape box, to assess memory based on distal environmental room cues. Another probe trial was conducted 30 days after the last training session to evaluate memory retention. The time spent around the target hole was recorded in these probe tests by the software.

#### T-maze test

##### Spontaneous alternation test

The spontaneous alternation test was conducted using an automated T-maze apparatus (O'HARA & Co., Tokyo, Japan). It was constructed of white plastic runways with walls 25 cm high. The maze was partitioned off into 6 areas by sliding doors that could be opened downward. The stem of the T was composed of area S2 (13 × 24 cm), and the arms of the T were composed of areas A1 and A2 (11.5 × 20.5 cm). Areas P1 and P2 were the connecting passageways from the respective arm (area A1 or A2) to the start compartment (area S1). Mice were subjected to a forced alternation protocol (see below) for 5 days (one session consisting of 10 trials per day; cut-off time, 50 min). Each trial had first and second runs. On the sample run, the mouse was forced to choose one of the arms of the T (area A1 or A2). After the mouse stayed more than 10 s, the door that separated the arm (area A1 or A2) and the connecting passageway (area P1 or P2) would be opened, and the mouse could return to the starting compartment (area S1) via the connecting passageway. The mouse was then given a 3 s delay in area S1, followed by a free choice between both T arms. The correct response was choosing the other arm that had not been chosen on the first trial of the pair. The location of the sample arm (left or right) was varied pseudo-randomly across trials using the Gellermann schedule so that mice received equal numbers of left and right presentations. A variety of fixed extra-maze clues surrounded the apparatus. On days 6–8, a delay (3, 10, 30, or 60 s) was applied after the sample trial. Data acquisition, control of sliding doors, and data analysis were performed by ImageTM software (see Section, “Data analysis”).

##### Forced alternation test using food reward

This test was conducted as previously described (Shoji et al., 2012). The forced alternation test used an automated T-maze apparatus (O'HARA & Co., Tokyo, Japan). The end of each arm was equipped with a pellet dispenser that could provide a food reward (a sucrose pellet). The pellet sensors were able to record pellet intake by the mice automatically. One week before the pre-training, mice were deprived of food until their body weight was reduced to 80–85% of their initial weight. Mice were kept on a maintenance diet throughout the forced alternation and left-right discrimination tests in the T-maze. Before the first session, mice were subjected to 30-min habituation session, during which they were allowed to freely explore the T-maze with all doors open and both arms baited with food. On the day after the habituation session, mice were subjected to a forced alternation protocol for 5 days (one session consisting of 10 trials per day; cut-off time, 50 min). Each trial had first and second runs. On the forced run, the mouse was forced to choose one of the arms of the T (area A1 or A2), and received the reward at the end of the arm. After the mouse either consumed the pellet or stayed more than 30 s without consuming the pellet (defined as an “Omission Error”), the door that separated the arm (area A1 or A2) and the connecting passageway (area P1 or P2) was opened and the mouse could return to the starting compartment (area S1) via the connecting passageway. In this way, the potential stress was reduced compared to the traditional forced alternation paradigm in which the human experimenter brings the mouse back to the start box by hand. The mouse was then given a 3 s delay in area S1, followed by a free choice between both T arms, and was rewarded for choosing the other arm that had not been chosen on the first trial of the pair. If the mouse failed to eat the pellet within 30 s, the response was recorded as an “Omission Error”. Choosing the incorrect arm resulted in no reward and confinement to the arm for 10 s. The location of the sample arm (left or right) was varied pseudo-randomly across trials using the Gellermann schedule so that mice received equal numbers of left and right presentations. A variety of fixed extra-maze clues surrounded the apparatus. On days 6–12, a delay (3, 10, 30, 60, or 120 s) was applied after the sample trial. Data acquisition, control of sliding doors, and data analysis were performed by ImageTM software (see Section, “Data analysis”).

##### Left-right discrimination test

This test was conducted as previously described (Shoji et al., 2012). The mice were placed in the stem of the T-maze. The door leading to the straight alley was opened and the mice were able to freely choose either the right or left arm of the T-maze. A sucrose pellet was always delivered to the food tray within one of the arms, which was the goal arm. Mice had to learn to enter the goal arm. If a mouse chose the goal arm, it was allowed to consume the food reward. The mouse was then allowed to return to the starting compartment. The correct arm was assigned to each mouse randomly. On day 8, the correct arm was changed to test reversal learning. Data acquisition, control of sliding doors, and data analysis were performed by ImageTM software (see Section, “Data analysis”).

#### Tail suspension test

The tail suspension test was performed for a 10-min test session. Mice were suspended 30 cm above the floor of a white plastic chamber (31 × 41 × 41 cm) (O'HARA & Co., Tokyo, Japan) in a visually isolated area by adhesive tape placed ~1 cm from the base of the tail, and behavior was recorded over a 10-min test period. Images were captured at 2 frame per s. Similar to the Porsolt forced swim test, immobility was judged by the application program according to a certain threshold. Immobility lasting for less than 2 s was not included in the analysis. Data acquisition and analysis were performed automatically, using ImageTS/PS software (see Section, “Data analysis”).

#### Contextual and cued fear conditioning test

A contextual and cued fear conditioning test was conducted as previously described (Shoji et al., 2014). Each mouse was placed in a transparent acrylic chamber (33 × 25 × 28 cm) with a stainless-steel grid floor (0.2 cm diameter, spaced 0.5 cm apart) (O'HARA & Co., Tokyo, Japan) illuminated at 100 lx, and allowed to explore freely for 2 min. A 55 dB white noise, which served as the conditioned stimulus (CS), was presented for 30 s, followed by a mild (2 s, 0.3 mA) foot shock, which served as the unconditioned stimulus (US). Two more CS-US pairings were presented with a 2-min inter-stimulus interval. Context test was conducted 24 h after conditioning in the same chamber. Cued test with altered context was conducted after conditioning using a triangular box (33 × 29 × 32 cm) made of white opaque Plexiglas, which was located in a different room. Thirty-five d after fear conditioning, mice were presented with the tone alone for 15 min in the altered context (extinction training phase). Twenty-four h after extinction training, mice were presented with the tone alone in the altered context (extinction test phase). The test chamber was illuminated at 30 lx. Data acquisition, control of stimuli (i.e., white noises and shocks), and data analysis were performed automatically, using ImageFZ software (see Section, “Data analysis”). Images were captured at 1 frame per s. For each pair of successive frames, the amount of area (pixels) by which the mouse moved was measured. When this area was below a certain threshold (i.e., 30 pixels), the behavior was classified as “freezing.” When the amount of area equaled or exceeded the threshold, the behavior was classified as “non-freezing.” The optimal threshold (amount of pixels) used to classify freezing was determined by adjusting it to the amount of freezing measured by human observation. “Freezing” that lasted less than the defined time threshold (i.e., 2 s) was not included in the analysis. The parameters were constant for all mice assessed.

#### Social interaction test in home cage

The social interaction monitoring system comprised the home cage and a filtered cage top with an infrared video camera (31 × 19 × 30 cm; 25 × 15 × 23.5 cm, inside dimensions). Two mice of the same genotype that had been housed separately were placed together in a home cage. To evaluate social interaction, their location was then monitored for 1 week. Output from the video camera was fed into a Windows computer. Images from each cage were captured at a rate of 1 frame per s. Social interaction was measured by counting the number of objects detected in each frame: two objects indicated that the mice were not in contact with each other, and one object indicated contact between the two mice. We also measured locomotor activity during these experiments by quantifying the number of pixels that changed between each pair of successive frames. Analysis was performed automatically using ImageHA software (see Section, “Data analysis”).

### Data analysis

Behavioral data were obtained automatically through applications based on the ImageJ program, and they were modified for each test by Tsuyoshi Miyakawa (available through O'HARA & Co., Tokyo, Japan). Statistical analysis was conducted using StatView (SAS Institute, Cary, NC, USA). Data were analyzed using two-tailed *t*-tests, Two-Way ANOVAs, Two-Way repeated measures ANOVAs, Mann–Whitney *U*-tests, or correlation *Z*-tests. Values in graphs are expressed as mean ± SEM or box plots (median, interquartile, and 90 and 10th percentiles). We defined “study-wide significance” as the statistical significance that survived Bonferroni correction for 89 indices employed in the behavioral test battery. “Nominal significance” was defined as the one that achieved a statistical significance in an index (*p* < 0.05) but did not survive this correction.

## Results

### General characterization of BARP KO mice

To study the physiological importance of BARP and its *in vivo* effects on psychiatric disorders, we generated transgenic mice in which BARP expression was knocked out (Supplementary Figure 1). We confirmed the deficiency of BARP in BARP KO mice using Southern, Northern, and Western blot analyses and genomic PCR analysis (Supplementary Figures 1B–E). As expected from the targeting construct for generation of BARP deficient mice (Supplementary Figure 1A), neurons expressing the BARP protein were detected by β-galactosidase activity in the BARP KO mice (data not shown). The regions that showed positive X-gal staining in the brains of BARP KO mice were the cortex, hippocampus, and cerebellum. This expression pattern is consistent with a previous report (Béguin et al., 2014).

**Figure 1:**
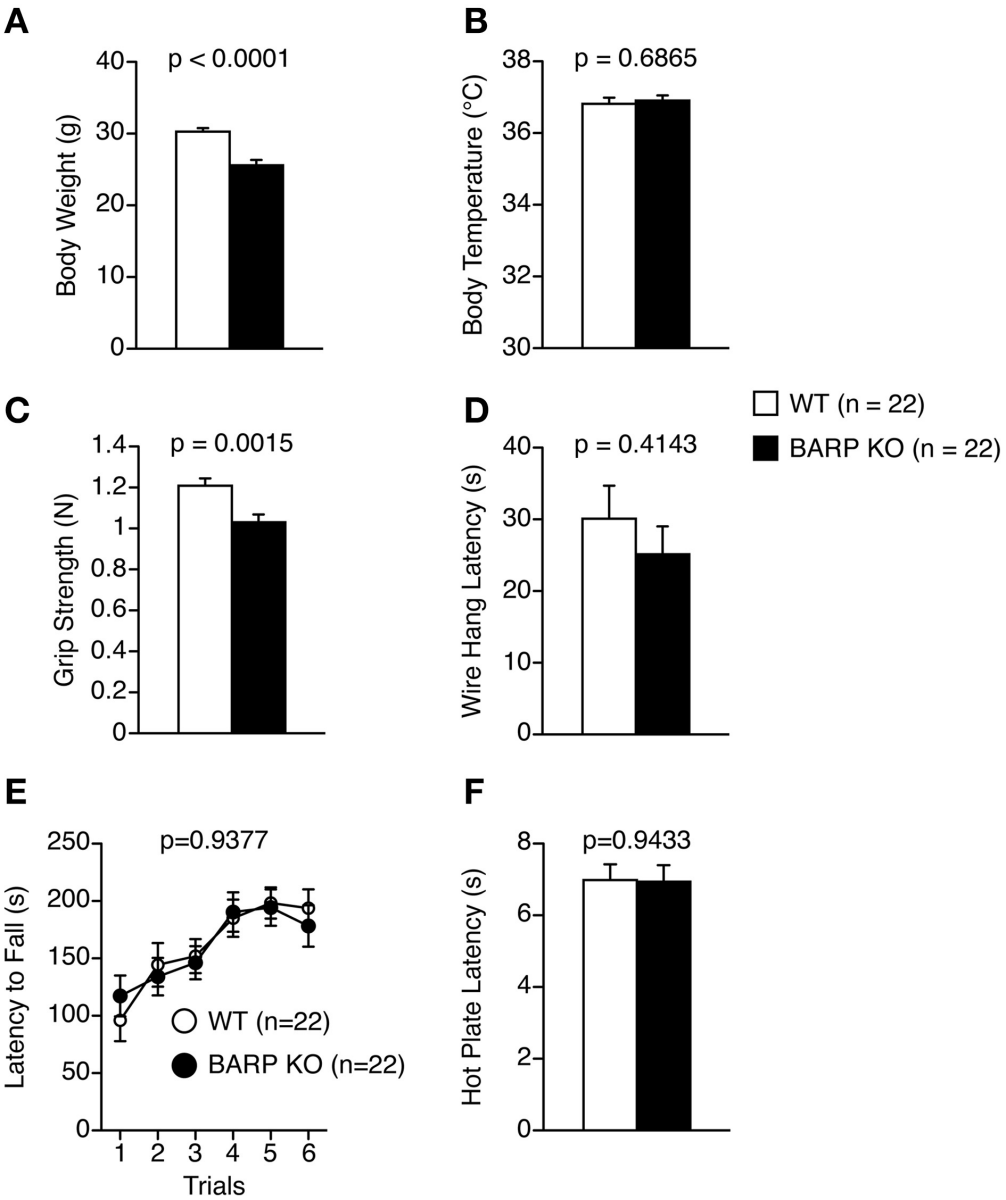
**General health and neurological screening, motor coordination/motor learning, and pain sensitivity in beta-anchoring and -regulatory protein (BARP), knockout (KO), mice, and wild type (WT) mice. (A)** Body weight. **(B)** Body temperature. **(C)** Grip strength score. **(D)** Wire hang latency. **(E)** Latency to fall off the rotating rod in the rotarod test. **(F)** Latency to the first fore- or hind-paw response in the hot plate test. Data represent the mean ± SEM. The *p*-values indicate a genotype effect in a *t*-test **(A–D,F)** or a Two-Way repeated measures ANOVA **(E)**.

We analyzed behaviors of BARP KO mice using a behavioral test battery (Powell and Miyakawa, 2006; Takao and Miyakawa, 2006a). The BARP KO mice weighed significantly less than their WT littermates (*t* = 5.308, *df* = 42, *p* < 0.0001) (Figure 1A). The difference reached study-wide significance. The BARP KO mice showed no obvious abnormalities on body temperature (Figure 1B), or on a gross inspection of their fur, whiskers, posture, righting reflex, whisker touch reflex, ear twitch reflex, and the response to key jangling (Table 1). The grip strength of BARP KO mice was nominally weaker than WT mice, suggesting that the BARP KO mice had muscle weakness and decreased motivation (*t* = 3.392, *df* = 42, *p* = 0.0015) (Figure 1C). In the wire hang test, there was no significant difference in the latency to fall off the wire grid between BARP KO and WT mice (Figure 1D). There were no significant differences in motor coordination (rotarod test, Figure 1E) and pain sensitivity (hot plate test, Figure 1F) compared with WT mice.

**Table 1:** **Summary of a comprehensive behavioral test battery for beta-anchoring and–regulatory protein (BARP) knockout (KO) mice in comparison with wild type (WT) mice**.

**Test**	**Measure**	**Alteration from WT**
General health, neurological, and wire hang	Gross inspection of their fur	→
	Whiskers	→
	Posture	→
	Righting reflex	→
	Whisker touch reflex	→
	Ear twitch reflex	→
	Response to key jangling	→
	Body weight	↓↓
	Body temperature	→
	Grip Strength	↓
	Wire hang time	→
Rotarod	Latency to fall	→
Hot plate	Latency	→
Light/dark transition	Transitions	↓
Elevated plus maze	Entries into open arms	→
Open field	Total distance	↓ (0–60 min)
	Vertical activity	↓↓
	Center time	↓ (30 and 55 min time points)
	Stereotypic Counts	↓↓
Porsolt forced swimming	Immobility	→
Tail suspension	Immobility	→
Social interaction (novel environment)	Total duration of contacts	→
	Number of contacts	→
	Total duration of active contact	→
	Mean duration / Contact	→
	Distance traveled	→
Social interaction (Crawley ver.)	Time spent in chamber	See Results
	Time spent around cage	See Results
Social interaction (home cage)	Mean number of objects	↓
	Activity level during overall period	↓↓
	Activity level during dark period	↓↓
	Activity level during light period	↓
Prepulse inhibition	Acoustic startle response	↓↓
	Prepulse inhibition	↑
Fear conditioning test	Freezing in during conditioning	See Results
	Freezing during the context test	See Results
	Freezing during cued test with altered context	See Results
Barnes maze	Latency to enter into the target hole	→
	Probe test (1 day after training)	→
	Probe test (30 days after training)	→
T-maze (spontaneous test)	Correct responses during training	↑ (4 and 5 sessions)
	Correct responses in the delay session	↑
	Latency to complete a session	↑↑
T-maze (forced alteration test)	Correct responses during training	→
	Correct responses in the delay session	↑ (120 s)
	Latency to complete a session	→
T-maze (left-right discrimination test)	Correct responses (reversal)	↑
	Latency to complete a session	→

*Symbols: →, no significant difference (p > 0.05); ↑↑, increase that reached study-wide significance; ↓↓, decrease that reached study-wide significance; ↑, nominally significant increase; ↓, nominally significant decrease; in comparison with WT littermates*.

### Decreased locomotor activity in BARP KO mice

**Figure 2:**
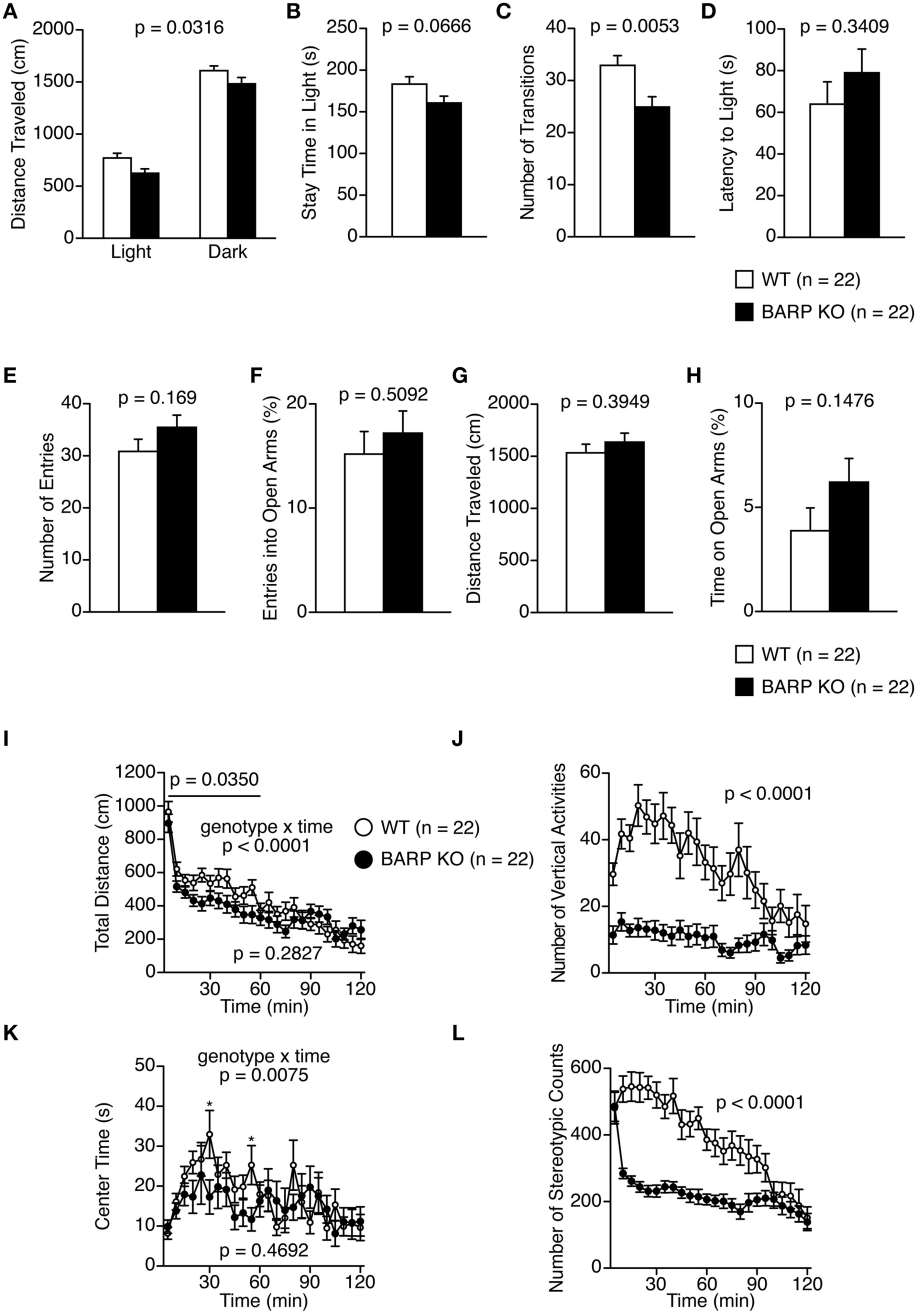
**Decreased locomotor activity in beta-anchoring and -regulatory protein (BARP) knockout (KO) mice. (A–D)** Light/dark transition test: the distance traveled in the light/dark compartments **(A)**, time spent in the light compartment **(B)**, number of light/dark transitions **(C)**, and latency to enter the light compartment **(D)** are shown. **(E–H)** Elevated plus maze test: the number of arm entries **(E)**, percentage of entries into open arms **(F)**, distance traveled **(G)**, and percentage of time spent in open arms **(H)** are shown. **(I–L)** Open field test: the total distance traveled **(I)**, vertical activity **(J)**, time spent in the center area **(K)**, and stereotypic behavior counts **(L)**. Data represent the mean ± SEM. The *p*-values indicate the genotype effect in a *t*-test **(B–H)** and a Two-Way repeated measures ANOVA **(A,I–L)**.

In the light/dark transition test, BARP KO mice showed a nominally significant decrease in total distance traveled in the light and dark chambers, and number of transitions compared with WT mice [genotype effect, *F*_(1, 42)_ = 4.945, *p* = 0.0316; genotype × time effect, *F*_(1, 42)_ = 0.100, *p* = 0.7536 and *t* = 2.939, *df* = 42, *p* = 0.0053, respectively] (Figures 2A,C). However, BARP KO and WT mice showed similar stay time in the light chamber (*t* = 1.883, *df* = 42, *p* = 0.0666), and similar latency to the first transition (*t* = 0.963, *df* = 42, *p* = 0.3409) (Figures 2B,D).

In the elevated plus maze test, no significant differences between BARP KO and WT mice were found in any of the parameters examined (Figures 2E–H).

In the open-field test, BARP KO mice showed a nominally significant decrease in distance traveled compared with WT mice in the initial 60 min [Figure 2I; total, genotype effect, *F*_(1, 42)_ = 1.184, *p* = 0.2827; genotype × time effect, *F*_(23, 966)_ = 3.087, *p* < 0.0001; 0–60 min, genotype effect, *F*_(1, 42)_ = 4.747, *p* = 0.0350]. Vertical activity and stereotypic behavior counts were significantly reduced in BARP KO mice compared with WT mice [*F*_(1, 42)_ = 25.380, *p* < 0.0001, and *F*_(1, 42)_ = 26.134, *p* < 0.0001, respectively] (Figures 2J,L). These differences reached study-wide significance. BARP KO mice showed a nominally significant decrease in time spent in the center of the open field apparatus at the 30 and 55 min time points [total, genotype effect, *F*_(1, 42)_ = 0.534, *p* = 0.4692; genotype × time effect, *F*_(23, 966)_ = 1.877, *p* = 0.0075; 30 min, simple main effect, *F*_(1, 42)_ = 4.542, *p* = 0.0390; 55 min, simple main effect, *F*_(1, 42)_ = 5.738, *p* = 0.0211] (Figure 2K).

### Normal depression-like behavior in BARP KO mice

**Figure 3:**
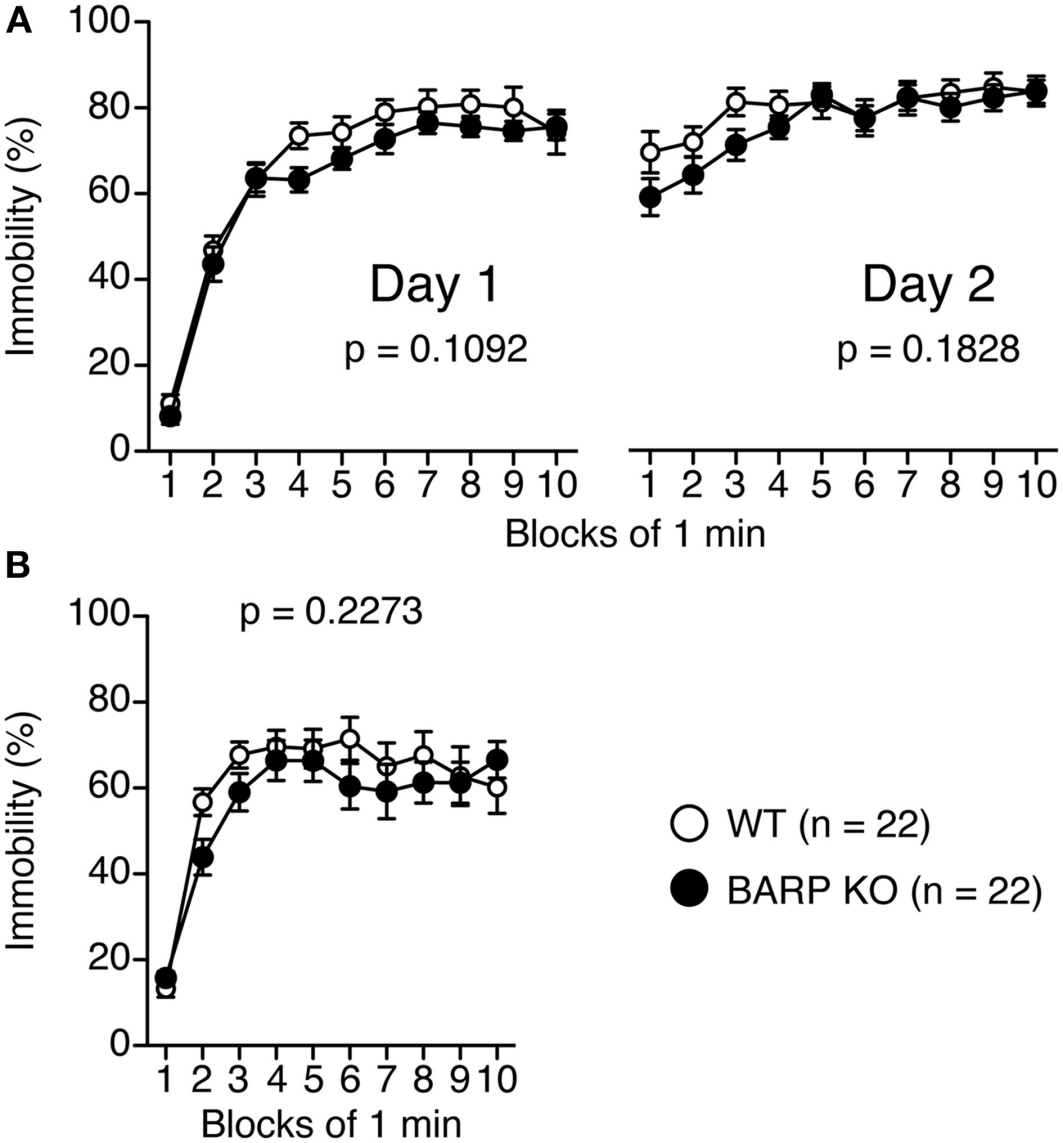
**Normal depression-like behavior in beta-anchoring and -regulatory protein (BARP) knockout (KO) mice**. Immobility on day 1 and day 2 for BARP KO and wild type (WT) mice in the Porsolt forced swim test **(A)**. Immobility time for BARP KO and WT mice in the tail suspension test **(B)**. Data represent the mean ± SEM. The *p*-values indicate a genotype effect in a Two-Way repeated measures ANOVA.

For the Porsolt forced swim test, there were no significant effects of genotype on the percentage of immobility time on day 1 [genotype effect, *F*_(1, 42)_ = 2.679, *p* = 0.1092; genotype × time effect, *F*_(9, 378)_ = 0.663, *p* = 0.7425] and on day 2 [genotype effect, *F*_(1, 42)_ = 1.835, *p* = 0.1828; genotype × time effect, *F*_(9, 378)_ = 1.054, *p* = 0.3962] (Figure 3A). In the tail suspension test (Figure 3B), no significant genotype effect was found for the percentage of immobility time [genotype effect, *F*_(1, 42)_ = 1.501, *p* = 0.2273; genotype × time effect, *F*_(9, 378)_ = 1.000, *p* = 0.4392].

### Nominally significant increase in sociability in BARP KO mice

**Figure 4:**
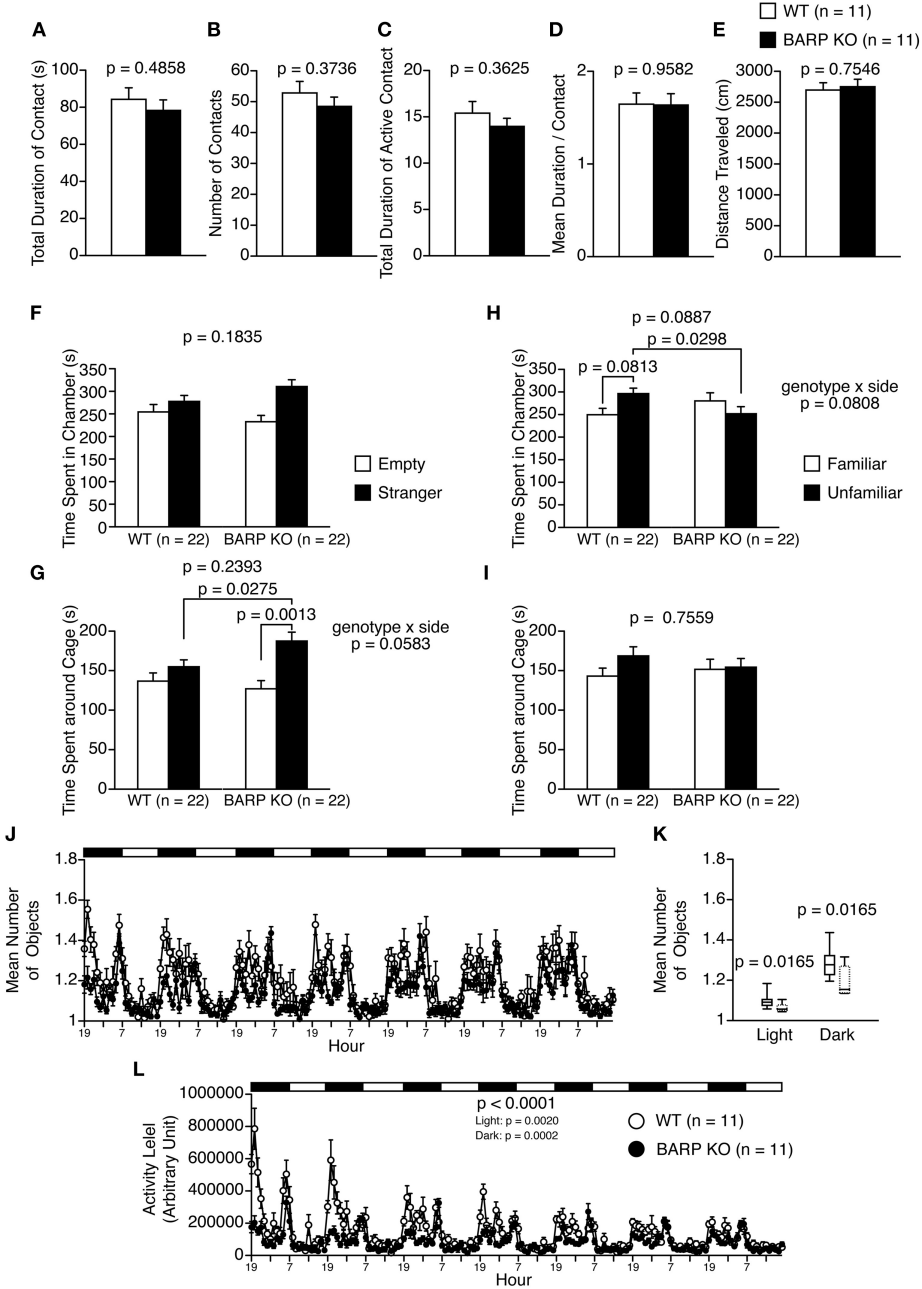
**Nominally significant increase in sociability in beta-anchoring and -regulatory protein (BARP) knockout (KO) mice. (A–E)** Social interaction test: the total duration of contacts **(A)**, number of contacts **(B)**, total duration of active contacts **(C)**, mean duration of each contact **(D)**, and total distance traveled **(E)** are shown. **(F–I)** Sociability test **(F,G)** and social novelty preference test **(H,I)**: the time spent in chamber in the sociability test **(F)**, time spent around cage in the sociability test **(G)**, time spent in chamber in the social novelty preference test **(H)**, and time spent around cage in the sociability test **(I)**. In the sociability test, the time spent in the chamber with the empty cage (empty side) and with the cage containing a stranger (stranger side) are shown, and the time spent in the vicinity of the empty cage (empty side) vs. the cage containing a stranger (stranger side) are shown. In the preference for social novelty test, the time spent in the chamber with the cage containing a stranger (unfamiliar side) and with the cage containing a familiar (familiar side) are shown, and the time spent in the vicinity of the cage containing a stranger (unfamiliar side) and the cage containing a familiar (familiar side) are shown. **(J–L)** 24-h home cage social interaction test: mean number of objects detected **(J)**, box plot distributions of mean number of objects detected (WT, solid line; BARP KO, dashed line) **(K)**, and activity level **(L)** over 7 days. Data are shown as mean ± SEM or in box plots (median, interquartile, and 90 and 10th percentiles). The *p*-values indicate the genotype effect in a *t*-test **(A–I)**, a Mann–Whitney *U*-test **(K)** and a Two-Way repeated measures ANOVA **(L)**.

In the social interaction test, there were no significant differences between BARP KO and WT mice in their total duration of contacts (*t* = 0.710, *df* = 20, *p* = 0.4858) (Figure 4A), number of contacts (*t* = 0.910, *df* = 20, *p* = 0.3736) (Figure 4B), total duration of active contacts (*t* = 0.932, *df* = 20, *p* = 0.3625) (Figure 4C), mean duration per contact (*t* = 0.053, *df* = 20, *p* = 0.9582) (Figure 4D), or distance traveled (*t* = 0.317, *df* = 20, *p* = 0.7546) (Figure 4E).

The three-chamber social approach test consists of a sociability test and a social novelty preference test, defined by the time spent around a wire cage containing a stranger mouse vs. an empty cage in the sociability test, and an unfamiliar mouse vs. a familiar mouse in the social novelty preference test (Moy et al., 2004). In the sociability test, the time spent in chamber was statistically indistinguishable between BARP KO and WT [genotype effect, *F*_(1, 42)_ = 1.829, *p* = 0.1835; genotype × side, *F*_(1, 42)_ = 1.788, *p* = 0.1883] (Figure 4F). A Two-Way repeated measures ANOVA showed no significant genotype effect, while there was marginally significant interaction in time around cage [genotype effect, *F*_(1, 42)_ = 1.425, *p* = 0.2393; genotype × side interaction, *F*_(1, 42)_ = 3.790, *p* = 0.0583]. Paired and unpaired *t*-tests showed that there were nominally significant differences in time spend around cage in BARP KO (*t* = 3.724, *df* = 21, *p* = 0.0013, stranger side vs. empty cage side) (Figure 4G), and in time spent around cage with stranger between genotypes (*t* = 2.284, *df* = 42, *p* = 0.0275, BARP KO vs. WT) (Figure 4G). In the social novelty preference test, a Two-Way repeated measures ANOVA showed no significant genotype effect in time spent in chamber [*F*_(1, 42)_ = 3.036, *p* = 0.0887]. Since there was marginally significant interaction in time spent in chamber [*F*_(1, 42)_ = 3.200, *p* = 0.0808], we conducted unpaired and paired *t*-tests on time spent in chambers. There was a nominally significant difference between genotypes in time spent in chamber of unfamiliar side (*t* = 2.250, *df* = 42, *p* = 0.0298, BARP KO vs. WT) (Figure 4H). The time spent in each chamber was statistically indistinguishable between the familiar and unfamiliar sides in BARP KO mice (*t* = 0.851, *df* = 21, *p* = 0.4042) (Figure 4H), while WT mice tended to spend more time in the chamber of unfamiliar side than that of familiar side (*t* = 1.831, *df* = 21, *p* = 0.0813) (Figure 4H). There was no statistically significant genotype effect nor genotype × side interaction in time spent around cage [genotype effect, *F*_(1, 42)_ = 0.098, *p* = 0.7559; genotype × side interaction, *F*_(1, 42)_ = 0.728, *p* = 0.3985] (Figure 4I). To avoid false-positives caused by the multiple statistical tests used, Bonferroni correction was applied to the results in the three-chamber social approach test (the adjusted *p*-value at the 0.05 significance level for 16 indices was 0.003125). After the correction, results remained significant for time spent around the cage by BARP KOs in the sociability test (*t* = 3.724, *df* = 21, *p* = 0.0013, stranger side vs. empty cage side) (Figure 4G).

It should be noted that the time spent by WT mice around a stranger mouse was similar to that spent around an empty cage (Figure 4G). In this study, a habituation session was performed in the apparatus for 10 min the day before the sociability test, while it is conventionally conducted immediately before the sociability test (Moy et al., 2004). The time spent around a stranger cage could be determined by approach-avoidance conflict and the protocol we used could have shifted the conflict toward avoidance behavior by increasing the relative novelty of the environment.

The social interaction of BARP KO mice in the home cage under familiar conditions was observed over a 7 days period (Figures 4J,K). In the social interaction test in the home cage, the mean number of objects usually increases when mice are active and decreases when mice are sleeping in one place. BARP KO mice spent less time separated from each other than WT mice in the light (*p* = 0.0165) and dark periods (*p* = 0.0165) (Figures 4J,K). This result suggests that the duration of social interaction was increased in BARP KO mice compared with WT mice. In addition, locomotor activity was lower in BARP KO mice in the overall period [genotype effect, *F*_(1, 20)_ = 25.223, *p* < 0.0001; genotype × time interaction, *F*_(167, 3340)_ = 6.521, *p* < 0.0001], light period [*F*_(1, 20)_ = 12.578, *p* = 0.0020], and dark period [*F*_(1, 20)_ = 21.489, *p* = 0.0002] (Figure 4L). The difference in the overall period and dark period reached study-wide significance.

### Decreased acoustic startle response and nominally significant increase in PPI in BARP KO mice

**Figure 5:**
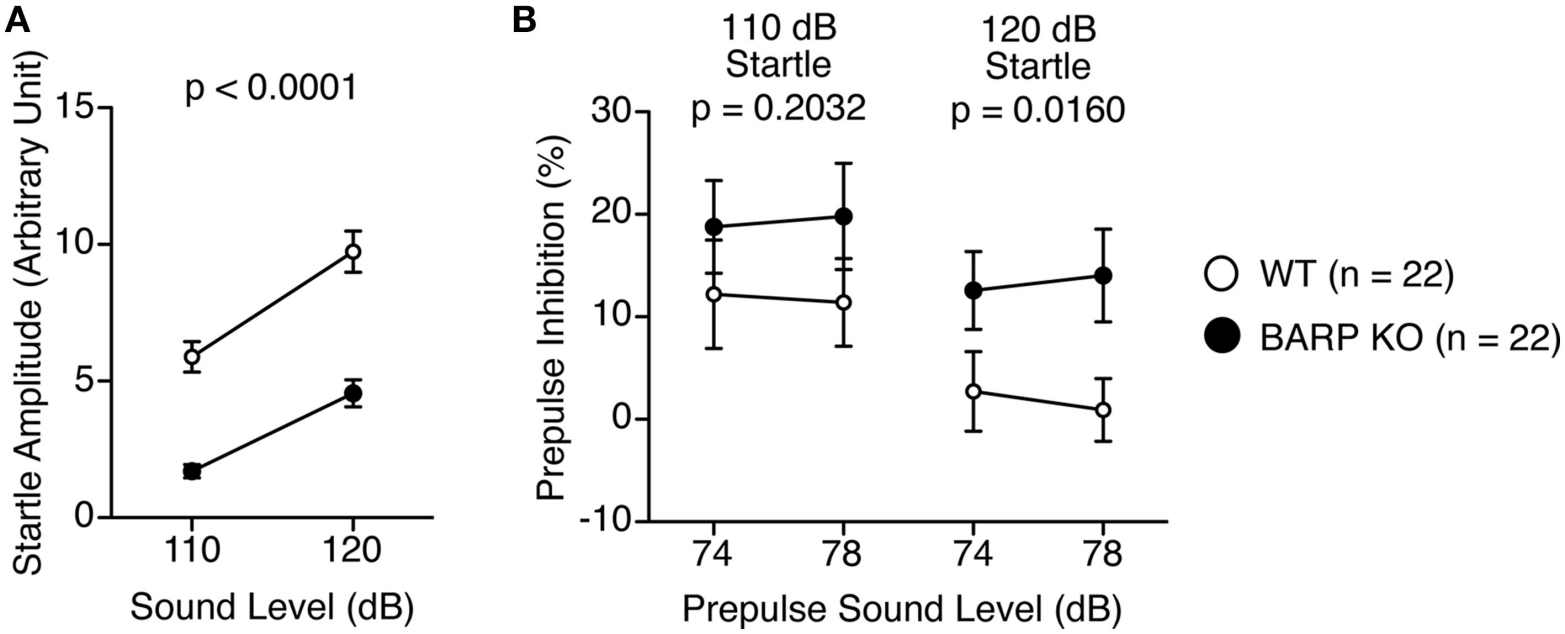
**Decreased acoustic startle response and nominally significant increase in prepulse inhibition (PPI) in beta-anchoring and -regulatory protein (BARP) knockout (KO) mice. (A)** Amplitude of the startle response. **(B)** Percentage of PPI. Data represent the mean ± SEM. The *p*-values indicate a genotype effect in a Two-Way repeated measures ANOVA.

The acoustic startle response of the BARP KO mice at 110 and 120 dB was significantly decreased [*F*_(1, 42)_ = 43.015, *p* < 0.0001], which reached study-wide significance (Figure 5A). PPI, whose decrease often reflects psychiatric disturbance (Swerdlow et al., 2000), was significantly increased at 120 dB in the BARP KO mice [genotype effect, *F*_(1, 42)_ = 1.671, *p* = 0.2032; genotype × prepulse sound level effect, *F*_(1, 42)_ = 0.062, *p* = 0.8042 in startle response at 110 dB; genotype effect, *F*_(1, 42)_ = 6.296, *p* = 0.0160 in startle response at 120 dB] (Figure 5B). Since it is possible that the apparent increase in PPI was due to a secondary effect of a decreased acoustic startle response in BARP KO mice, we examined the correlation between startle amplitude and PPI. There was no significant correlation between startle amplitude and PPI [WT: startle amplitude (110 dB) and PPI (74–110 dB), *r* = 0.176, *p* = 0.4377, startle amplitude (110 dB) and PPI (78–110 dB), *r* = 0.051, *p* = 0.8277, startle amplitude (120 dB) and PPI (74–120 dB), *r* = 0.202, *p* = 0.3716, startle amplitude (120 dB) and PPI (78–120 dB), *r* = 0.175, *p* = 0.4417; BARP KO: startle amplitude (110 dB) and PPI (74–110 dB), *r* = −0.147, *p* = 0.5184, startle amplitude (110 dB) and PPI (78–110 dB), *r* = 0.149, *p* = 0.5134, startle amplitude (120 dB) and PPI (74–120 dB), *r* = 0.110, *p* = 0.6293, startle amplitude (120 dB) and PPI (78–120 dB), *r* = 0.127, *p* = 0.5789) (Supplementary Figures 3A–D). Thus, it is unlikely that the apparent increase in PPI was due to a secondary effect of a decreased acoustic startle response in BARP KO mice.

### Nominally significant increase in suppression ratio during pre-tone period at 1 d after conditioning in BARP KO mice

**Figure 6:**
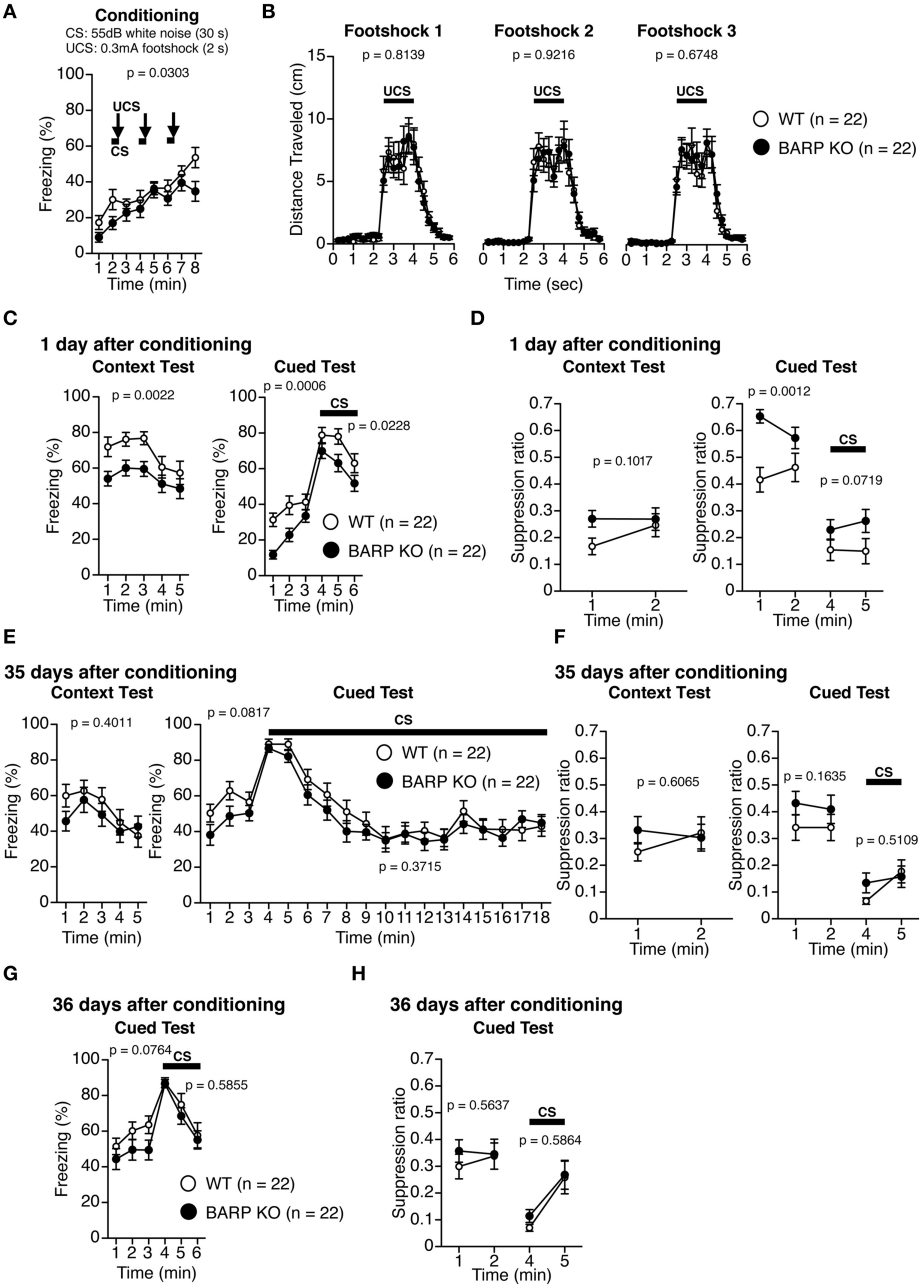
**Nominally significant increase in suppression ratio during pre-tone period at 1 day after conditioning in beta-anchoring and -regulatory protein (BARP) knockout (KO) mice. (A)** Freezing during conditioning. **(B)** Distance traveled during and after each foot shock in the training phase. **(C)** Freezing during the context test (left) and the cued test (right) 1 day after conditioning. **(D)** The suppression ratio in the context (left) and cued tests (right) 1 day after conditioning. **(E)** Freezing during the context test (left) and the cued test (right) 35 days after conditioning. **(F)** The suppression ratio in the context (left) and cued tests (right) 35 days after conditioning. **(G)** The cued test 36 days after conditioning. **(H)** The suppression ratio in the cued test 36 days after conditioning. Data represent the mean ± SEM. Data were analyzed using a Two-Way repeated measures ANOVA, and the *p*-values indicate a genotype effect.

Pavlovian fear conditioning forms a strong association between a CS and an aversive US that can trigger stereotypic fear responses such as freezing (Maren, 2001; Pape and Pare, 2010). On the first day, BARP KO and WT mice were placed into a chamber and conditioned with a tone (the CS) paired with a foot shock (the US) (conditioning). On the second day, the mice were returned to the chamber and the incidences of freezing were examined in the absence of the tone and foot shock (context test). Next, conditioned mice were placed in a novel chamber, where freezing behavior was measured after the presentation of the tone without the foot shock (cued test). During the conditioning period, BARP KO mice showed lower levels of freezing before, during, and after foot shocks [*F*_(1, 42)_ = 5.026, *p* = 0.0303] (Figure 6A). There was no significant difference in the distance traveled during and after each foot shock in the conditioning period [foot shock 1, genotype effect, *F*_(1, 42)_ = 0.056, *p* = 0.8139; genotype × time effect, *F*_(22, 924)_ = 0.504, *p* = 0.9726; foot shock 2, genotype effect, *F*_(1, 42)_ = 0.01, *p* = 0.9216; genotype × time effect, *F*_(22, 924)_ = 0.47, *p* = 0.9824; foot shock 3, genotype effect, *F*_(1, 42)_ = 0.179, *p* = 0.6748; genotype × time effect, *F*_(22, 924)_ = 0.422, *p* = 0.9914] (Figure 6B). To control for baseline activity, we classified the distance traveled during the first 2 min of training as “baseline activity” and used a suppression ratio [suppression ratio = (activity during testing)/(activity during baseline + activity during testing)] as a secondary index of fear (Anagnostaras et al., 2000; Frankland et al., 2004). In the context test 1 day after conditioning, BARP KO mice showed reduced levels of freezing, although the suppression ratio was indistinguishable between BARP KO and WT mice [freezing, genotype effect, *F*_(1, 42)_ = 10.69, *p* = 0.0022; genotype × time effect, *F*_(4, 168)_ = 0.506, *p* = 0.7310; suppression ratio, genotype effect, *F*_(1, 42)_ = 2.799, *p* = 0.1017; genotype × time effect, *F*_(1, 42)_ = 1.194, *p* = 0.2807] (Figures 6C,D). In the cued test with altered context 1 day after conditioning, BARP KO mice showed a nominally significant reduction in levels of freezing during pre-tone period [genotype effect, *F*_(1, 42)_ = 13.741, *p* = 0.0006] and during the CS [genotype effect, *F*_(1, 42)_ = 5.589, *p* = 0.0228] (Figure 6C). Suppression ratio during pre-tone period showed a nominally significant decrease in BARP KO [genotype effect, *F*_(1, 42)_ = 12.025, *p* = 0.0012], while the one during the CS was statistically indistinguishable between BARP KO and WT mice [*F*_(1, 42)_ = 3.408, *p* = 0.0719; genotype × time effect, *F*_(1, 42)_ = 0.386, *p* = 0.5379] (Figure 6D). In the context test 35 days after conditioning, there were no significant genotype effects nor genotype × time interactions in freezing [genotype effect, *F*_(1, 42)_ = 0.719, *p* = 0.4011; genotype × time effect, *F*_(4, 168)_ = 1.078, *p* = 0.3689] and in suppression ratio [genotype effect, *F*_(1, 42)_ = 0.269, *p* = 0.6065; genotype × time effect, *F*_(1, 42)_ = 1.975, *p* = 0.1672] (Figures 6E,F). In the cued test 35 days after conditioning, levels of freezing during pre-tone period [genotype effect, *F*_(1, 42)_ = 3.181, *p* = 0.0817; genotype × time effect, *F*_(2, 84)_ = 0.659, *p* = 0.5199] and during the CS [genotype effect, *F*_(1, 42)_ = 0.816, *p* = 0.3715; genotype × time effect, *F*_(14, 588)_ = 0.518, *p* = 0.9236] were statistically indistinguishable between BARP KO and WT (Figure 6E). There were no significant genotype effects nor genotype × time interactions in suppression ratio during pre-tone period [genotype effect, *F*_(1, 42)_ = 2.011, *p* = 0.1635; genotype × time effect, *F*_(1, 42)_ = 0.086, *p* = 0.7710] and during the CS [genotype effect, *F*_(1, 42)_ = 0.440, *p* = 0.5109; genotype × time effect, *F*_(1, 42)_ = 1.600, *p* = 0.2129] (Figure 6F).

To examine the role of BARP on cued fear extinction, we exposed WT and BARP KO mice to repeated non-reinforced CS presentations for 15 min at 35 days after fear conditioning (Figure 6E, right). A cued test with altered context was conducted 1 day after extinction training (Figures 6G,H). There were no significant genotype effects nor genotype × time interactions in freezing during pre-tone period [baseline, genotype effect, *F*_(1, 42)_ = 3.300, *p* = 0.0764; genotype × time effect, *F*_(2, 84)_ = 0.372, *p* = 0.6905] or during the CS [genotype effect, *F*_(1, 42)_ = 0.302, *p* = 0.5855; genotype × time effect, *F*_(2, 84)_ = 0.364, *p* = 0.6958]. We failed to detect the statistically significant differences in suppression ratio during pre-tone period [genotype effect, *F*_(1, 42)_ = 0.339, *p* = 0.5637; genotype × time effect, *F*_(1, 42)_ = 0.397, *p* = 0.5318] or during the CS [genotype effect, *F*_(1, 42)_ = 0.301, *p* = 0.5864; genotype × time effect, *F*_(1, 42)_ = 0.220, *p* = 0.6415].

### Normal acquisition and retention of spatial reference memory in BARP KO mice

**Figure 7:**
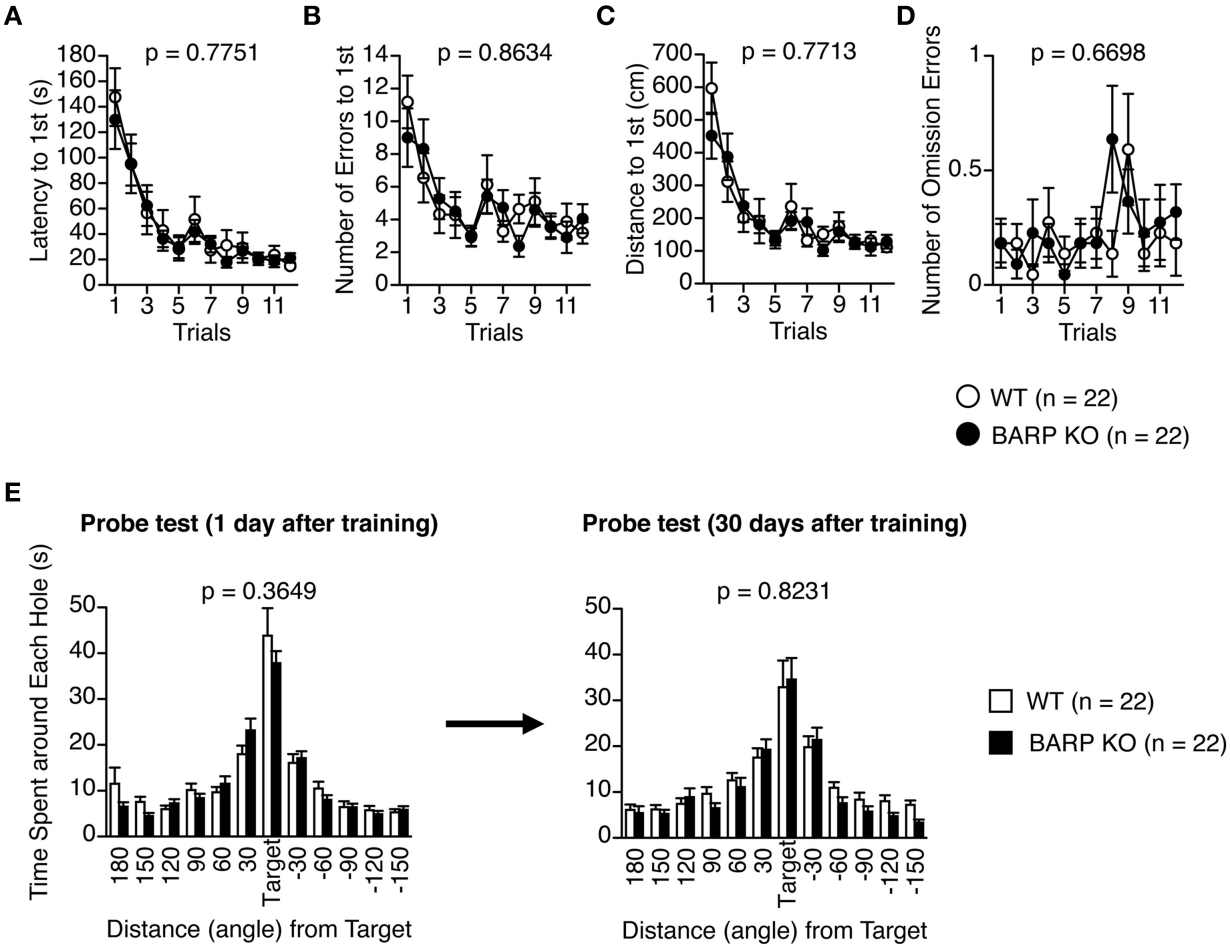
**Normal acquisition and retention of spatial reference memory in beta-anchoring and -regulatory protein (BARP) knockout (KO) mice. (A)** Latency to reach the target hole. **(B)** Number of errors before reaching the target hole. **(C)** Distance to reach the target hole. **(D)** Number of omission errors before reaching the target hole. Data were analyzed by Two-Way repeated measures ANOVA. Data are presented as means of three trials. The *p*-values indicate a genotype effect in a Two-Way repeated measures ANOVA. **(E)** Time spent around each hole in the probe trial conducted 24 h (left) and 1 month (right) after the last training session. The *p*-values indicate a genotype effect in a *t*-test.

In the Barnes maze test, there was no significant effect of genotype on the latency to find the target hole [*F*_(1, 42)_ = 0.083, *p* = 0.7751] (Figure 7A), the number of search errors made [*F*_(1, 42)_ = 0.030, *p* = 0.8634] (Figure 7B), the distance to reach the target hole during acquisition [*F*_(1, 42)_ = 0.086, *p* = 0.7713] (Figure 7C), or the number of omission errors [*F*_(1, 42)_ = 0.184, *p* = 0.6698] (Figure 7D), indicating normal acquisition of spatial reference memory in BARP KO mice. Probe trials where the escape box was removed were performed 24 h and 30 days after the last day of training. There were no significant differences between genotypes in the time spent around the target during these probe tests (*t* = 0.916, *df* = 42, *p* = 0.3649, and *t* = 0.225, *df* = 42, *p* = 0.8231, respectively) (Figure 7E).

### Nominally significant increase in working memory and flexibility in BARP KO mice

**Figure 8:**
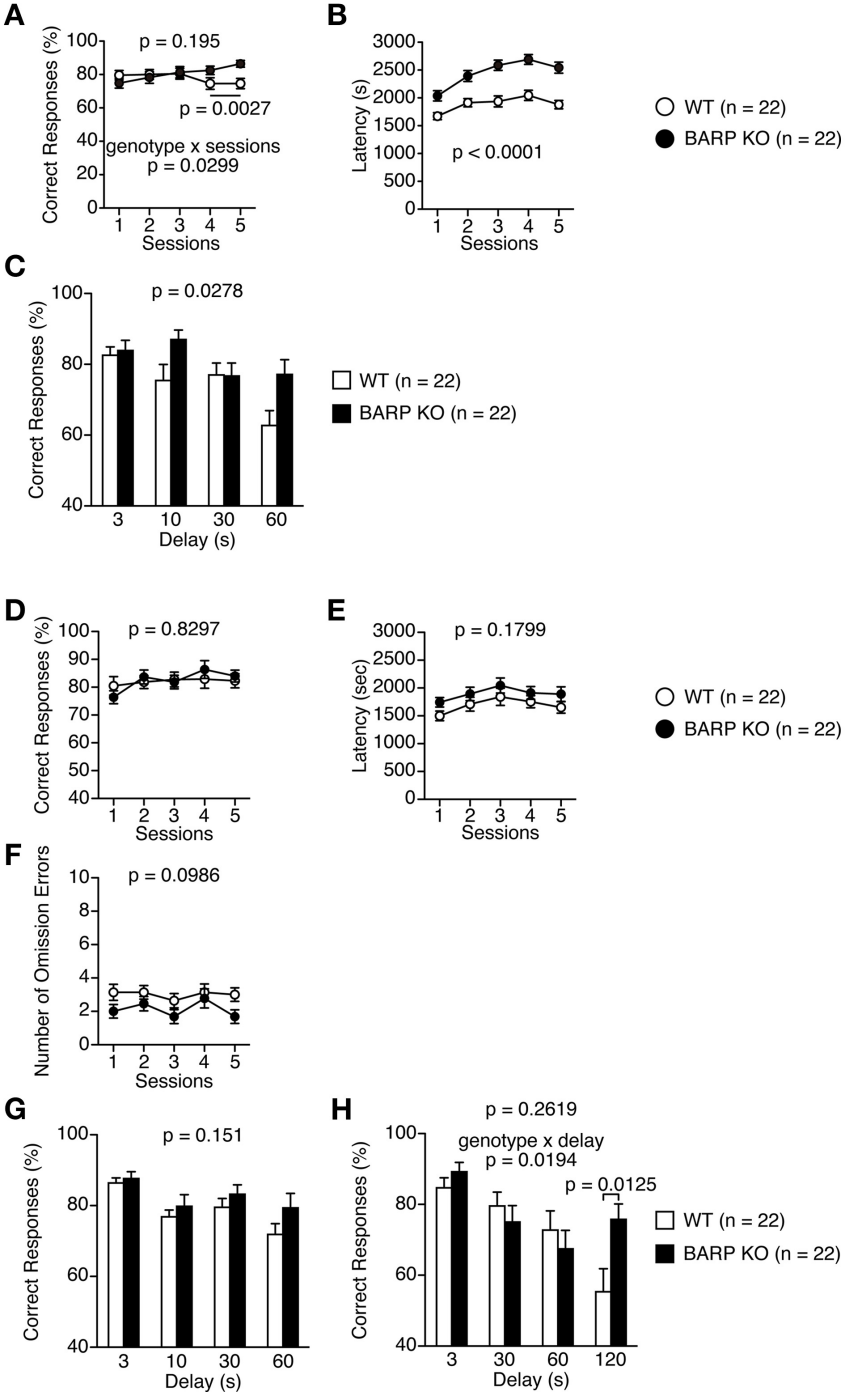
**Nominally significant increase in working memory in beta-anchoring and- regulatory protein (BARP) knockout (KO) mice. (A–C)** T-maze spontaneous alternation test: the percent correct responses **(A)** and latency **(B)** in the training session, and correct responses in the delay session **(C)** in BARP KO and wild type (WT) mice. **(D–H)** T-maze forced alternation test: the percent correct responses **(D)**, latency **(E)**, and omission errors **(F)** in the training session, and correct responses in the delay session **(G,H)** in BARP KO and WT mice. Data represent the mean ± SEM. Data were analyzed using a Two-Way repeated measures ANOVA and a *t*-test. The *p*-values indicate a genotype effect.

Mice were subjected to the spontaneous alteration, forced alternation using food reward, and left-right discrimination tests in the T-maze. In the T-maze spontaneous alternation test, BARP KO and WT mice were subjected to five consecutive sessions (Figures 8A–C). One session consisted of 10 trials. Each trial had first and second runs. On the first run of each trial, mice were forced to choose one of the goal arms (the other being blocked by a removable door). During the second run in which the mice had to remember the location of the visited arm in the first run, both goal arms were open and the mouse was able to choose one arm freely. BARP KO mice showed increased correct responses compared with WT mice in sessions four and five [total, genotype effect, *F*_(1, 42)_ = 1.734, *p* = 0.195; genotype × time effect, *F*_(4, 168)_ = 2.751, *p* = 0.0299; sessions four and five, genotype effect, *F*_(1, 42)_ = 10.206, *p* = 0.0027] (Figure 8A). BARP KO mice exhibited longer latency to complete a session in spontaneous T-maze alteration test (*F*_(1, 42)_ = 28.733, *p* < 0.0001) (Figure 8B). This difference reached study-wide significance. With 10, 30, and 60 s delays between the forced and free choices, there was a significant difference in the percentage of correct choices between BARP KO and WT mice [genotype effect, *F*_(1, 41)_ = 5.202, *p* = 0.0278] (Figure 8C).

Next, mice were subjected to the T-maze forced alternation tests using food reward after food restriction. BARP KO and WT mice were subjected to daily training sessions consisting of five consecutive sessions where the mice had to remember the location of the previously visited arm, similar to the T-maze spontaneous alternation test, to get a food reward. During training sessions, there was no significant difference between BARP KO and WT mice (Figures 8D–F, Supplementary Figure 4B). With 10, 30, and 60 s delays between the forced and free choices, the number of correct choices was indistinguishable between BARP KO and WT mice [genotype effect, *F*_(1, 42)_ = 2.139, *p* = 0.151; genotype × delay effect, *F*_(3, 126)_ = 0.657, *p* = 0.5798] (Figure 8G). To increase the difficulty of the task, a delay period (3, 30, 60, or 120 s) was applied. Under these conditions, BARP KO mice showed a nominally significant increase in spatial working memory with the 120 s delay [genotype effect, *F*_(1, 42)_ = 1.293, *p* = 0.2619; genotype × delay effect, *F*_(3, 126)_ = 3.420, *p* = 0.0194; 120 s delay, genotype effect, *t* = 2.609, *df* = 42, *p* = 0.0125] (Figure 8H).

**Figure 9:**
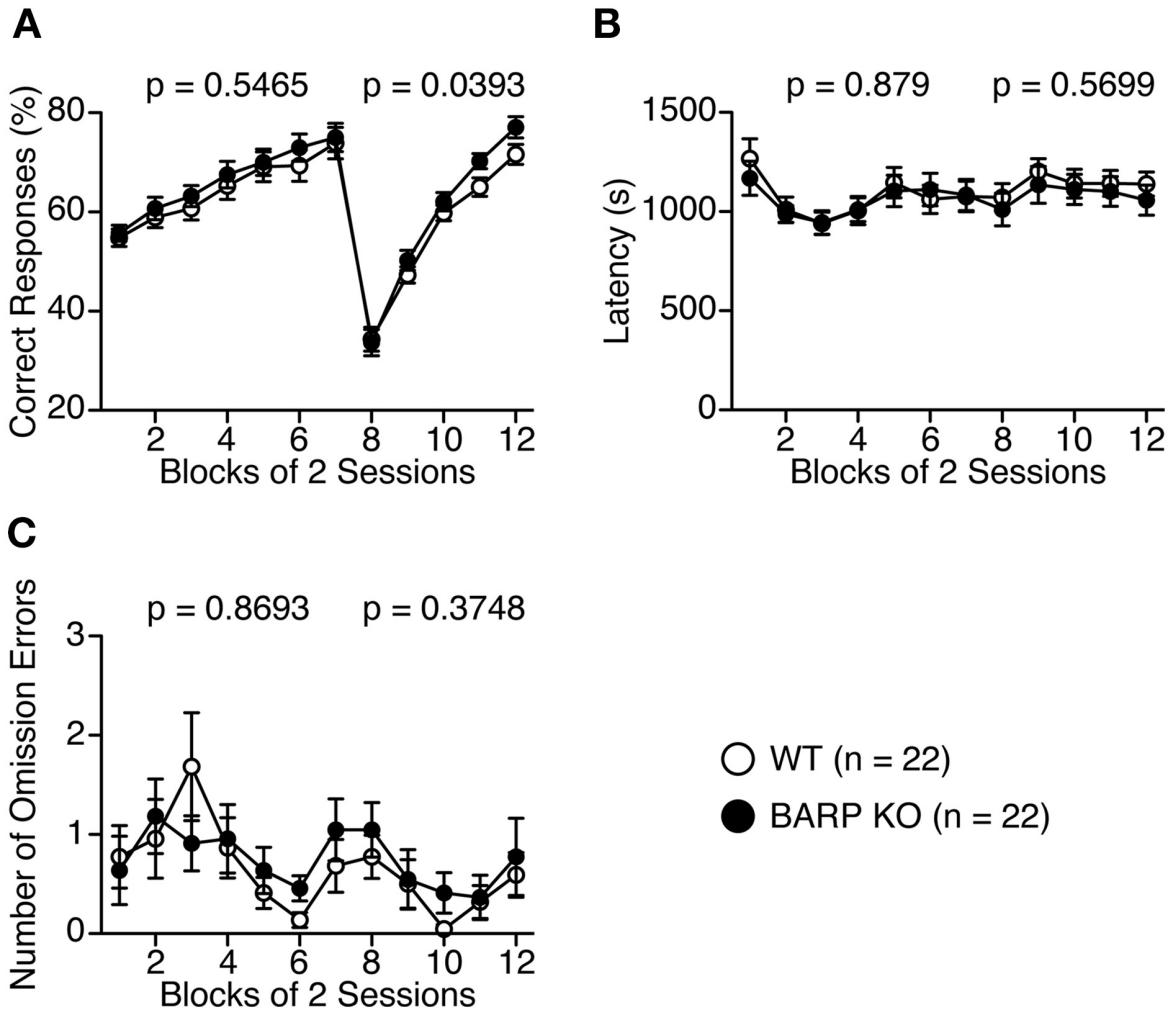
**Nominally significant decrease in a perseveration tendency of beta-anchoring and -regulatory protein (BARP) knockout (KO) mice in the T-maze left-right discrimination test**. Percent correct responses **(A)**, latency **(B)**, and omission errors **(C)** in BARP KO and wild type (WT) mice. The baited arm was changed to the opposite side from session eight onwards. Data represent the mean ± SEM. Data were analyzed using a Two-Way repeated measures ANOVA and the *p*-values indicate a genotype effect.

We then performed the left-right discrimination test in the T-maze to investigate perseveration tendency (Figures 9A–C). In the first seven sessions, where the baited arm was fixed to one side, correct responses were indistinguishable between BARP KO and WT mice [genotype effect, *F*_(1, 42)_ = 0.370, *p* = 0.5465; genotype × blocks of two sessions effect, *F*_(6, 252)_ = 0.251, *p* = 0.9585] (Figure 9A). However, in the following 8 days, when the baited arm was changed to the opposite side, the correct choice percentage was significantly higher in the BARP KO than the WT mice [*F*_(1, 42)_ = 4.528, *p* = 0.0393] (Figure 9A), suggesting a nominally significant increase in flexibility in the mutant mice.

## Discussion

In this study, we found that BARP KO mice have hypo-locomotor activity as evidenced by decreased vertical activity, stereotypic counts in the open field test, and activity level in the home cage, and longer latency to complete a session in spontaneous T-maze alteration test, which reached “study-wide significance.” In addition, some suggestive evidence showed that BARP KO mice may have increased anxiety-like behavior and multiple behavioral phenotypes that are seemingly opposite to those seen in the mouse models of schizophrenia and its related disorders.

The most pronounced behavioral phenotype of BARP KO mice is a decreased locomotor activity. It has been reported that CaV2.2 KO mice display hyperactivity (Beuckmann et al., 2003). Considering that BARP down-regulates activities of VGCCs including CaV2.2 (Béguin et al., 2014), it is possible that this behavioral phenotype of BARP KO mice could be mediated through decreased negative regulation of CaV2.2 by the lack of BARP.

BARP KO mice also exhibited contrasting behavioral phenotypes to those typically seen in mouse models of schizophrenia and its related disorders. BARP KO mice showed increased working memory ability, PPI, and social interaction, and decreased locomotor activity and perseveration, which are opposite to those generally considered as core features of schizophrenia (Braff and Geyer, 1990; Crider, 1997; Elvevag and Goldberg, 2000; Powell and Miyakawa, 2006), though many of the behavioral results suggesting these phenotypes achieved only nominal statistical significance and require further replication. In addition, greatly reduced vertical activity and stereotypic behavior counts in the open-field test in the mice are also in contrast to the behavioral phenotypes of some mouse models of schizophrenia and its related disorders, such as dopamine transporter KO mice, dominant-negative Disrupted-in-Schizophrenia-1 transgenic mice, Schnurri-2 KO mice, forebrain-specific calcineurin KO mice, and α-CaMKII heterozygous KO mice (Giros et al., 1996; Miyakawa et al., 2003; Hikida et al., 2007; Yamasaki et al., 2008; Takao et al., 2013). Molecules related to Ca^2+^ signaling, such as calcineurin and α-isoform of Ca^2+^/calmodulin dependent protein kinase II (α-CaMKII), act downstream of VGCC activation (Catterall, 2011), which is negatively regulated by BARP. Schizophrenia model mice such as forebrain-specific calcineurin KO mice and α-CaMKII heterozygous KO mice show hyperactivity, decreased working memory, and decreased PPI (Miyakawa et al., 2003; Yamasaki et al., 2008). It is possible that the deficit in BARP induces abnormal Ca^2+^ signaling via these molecules, contributing to these behavioral phenotypes.

We obtained some results that suggest increased anxiety-like behavior in BARP KO mice. The mutants exhibited nominally significant reductions in the number of transitions in the light/dark transition test. The number of transitions is considered as a core anxiety measure (Crawley et al., 1997). The BARP KO mice also showed a nominally significant reduction in time spent in the center of the open field apparatus, which is considered as an index of anxiety-like behavior, at a few time points. Since the *p*-values did not reach study-wide significance, there is the possibility that these results could be just false-positive and replication of behavioral study is needed. The possible increase of anxiety in the mice should be also examined by measuring other indices of anxiety, such as stress hormone level in the blood and expression level of immediately early genes in the amygdala.

It has been reported that genes encoding VGCC subunits, especially CACNA1C, are associated with psychiatric disorders, such as schizophrenia and bipolar disorder (Sklar et al., 2002, 2011; Ferreira et al., 2008; Ripke et al., 2011, 2014). We compared the behavioral phenotypes of BARP KO mice with those of mice with genetic or pharmacological inactivation of CaV1.2 encoded by CACNA1C (Table 2). We expected that BARP KO mice may show behavioral phenotypes opposite to those of CACNA1C transgenic mice, since BARP down-regulates VGCC activity (Béguin et al., 2014). However, behavioral phenotypes of BARP KO mice were not in consentient with such initial expectation (Table 2). One possibility to explain this inconsistency is that the lack of VGCC activity regulation by BARP could be compensated for by other VGCC β subunit-interacting proteins, such as Kir/Gem, Rem, Rad, RIMs, Bassoon, and CAST (Béguin et al., 2001; Finlin et al., 2003; Kiyonaka et al., 2007, 2012; Uriu et al., 2010; Chen et al., 2011). Another possibility is that a battery of multiple behavioral tests was applied to the same mice in the present study, while only a few behavioral tests were conducted for a mouse in the previous studies on CACNA1C. As mice were subjected to various kinds of behavioral tests, the stress/experience of multiple different tests might have affected the results in this study.

**Table 2:** **Behavioral phenotypes in mice with genetic or pharmacological inactivation of Ca_V_1.2 and beta-anchoring and -regulatory protein (BARP) knockout (KO)**.

	**BARP KO**	**Hippocampus and cortex CACNA1C KO**	**Acute pharmacological blockade of CaV1.2**	**ACC-specific conditional CACNA1C KO**	**Constitutive CACNA1C heterozygous**	**Forebrain-specific conditional CACNA1C KO**	**PFC-specific CACNA1C knockdown**
**Anxiety-like behavior**	Decreased number of transitions in the LD	Normal in the OF	N/D	Normal in the EP, LD, and OF	Decreased time spent in open arms in the EP Normal in the OF and LD	Decreased time spent in the open arms in the EP, in the center in the OF, and in the light in the LD	Decreased time spent in open arms in the EP
**Memory**	Better performance in the T-maze	Decreased performance during the training phase and in the remote memory probe test in the Morris water maze	Decreased performance at 1 day after conditioning in in cued fear conditioning paradigms	Impaired observational fear learning Normal in NOR and predator exposure Normal at 1 day after conditioning in cued and contextual fear conditioning paradigms	N/D	N/D	N/D
**Other behavioral phenotypes**	See Table 1	Normal in the rotarod test	N/D	N/D	N/D	N/D	Normal in locomotor activity
**References**	Present study	Moosmang et al., 2005; White et al., 2008	Langwieser et al., 2010	Jeon et al., 2010	Lee et al., 2012	Lee et al., 2012	Lee et al., 2012

*Abbreviations: ACC, anterior cingulate cortex; EP, elevated plus-maze test; LD, light-dark transition test; N/D, not determined; NOR, novel object recognition; PFC, prefrontal cortex; PS, Porsolt forced swim test*.

BARP KO mice showed lower levels of basal freezing before experiencing foot shocks in the fear conditioning test, although they were hypoactive in the open field test and home cage. One possibility to explain this inconsistency is that the increased locomotor activity displayed by BARP KO mice could be due to this particular environment (e.g., the grid floor). In the context and cued tests 1 day after conditioning, BARP KO mice also showed decreased freezing, though differences in the suppression ratio failed to reach significance between BARP KO and WT mice during the context test and during cue presentation in the cued test, possibly due to the lower levels of basal freezing in BARP KO mice. However, suppression ratio during pre-tone period in the new context in the cued test was significantly greater in the BARP KO mice, suggesting that they may have a better ability of pattern separation and/or decreased generalized fear. It should be noted that impaired pattern separation is proposed as a cognitive deficit of the patients with schizophrenia (Tamminga et al., 2010).

Taken together, our study shows that BARP KO mice are hypoactive and may possibly have increased anxiety. Moreover, the mice showed multiple behavioral phenotypes that are opposite to those considered as the behavioral abnormalities related to schizophrenia, suggesting that they may serve as a unique tool for studying the mechanisms of psychiatric disorders and their endophenotypes. Since many of the results obtained in this study are statistically weak, replications of the results are needed. Hence, further evaluation of the molecular and physiological phenotypes of BARP KO mice could provide new insights into the role of BARP in higher brain functions.

### Conflict of interest statement

The authors declare that the research was conducted in the absence of any commercial or financial relationships that could be construed as a potential conflict of interest.

## Abbreviations

BARP: beta-anchoring and -regulatory protein
lx: lux
PPI: prepulse inhibition
s: second
VGCC: voltage-gated calcium channel.
