# Supplementary material for: Comprehensive behavioral analysis of voltage-gated calcium channel beta-anchoring and -regulatory protein knockout mice
Source: Front Behav Neurosci. 2015 Jun 16;9:141. doi: 10.3389/fnbeh.2015.00141 (PMC4468383; doi:10.3389/fnbeh.2015.00141)
Supplement: Supplementary file 1 [file Table1.PDF]

Supplementary Material

Comprehensive behavioral analysis of voltage-gated calcium channel beta-anchoring and -regulatory protein knockout mice

Akito Nakao[1], Takafumi Miki[2], Hirotaka Shoji[1,3], Miyuki Nishi[4], Hiroshi Takeshima[4], Tsuyoshi Miyakawa[1,3,5]*, Yasuo Mori[2]*

Akito Nakao and Takafumi Miki contributed equally to this work.

[1]Division of Systems Medical Science, Institute for Comprehensive Medical Science, Fujita Health University, Toyoake, Aichi, Japan
[2]Department of Synthetic Chemistry and Biological Chemistry, Graduate School of Engineering, Kyoto University, Kyoto, Kyoto, Japan
[3]Japan Science and Technology Agency (JST), Core Research for Evolutional Science and Technology (CREST), Kawaguchi, Saitama, Japan
[4]Department of Biological Chemistry, Graduate School of Pharmaceutical Sciences, Kyoto University, Kyoto, Kyoto, Japan
[5]Center for Genetic Analysis of Behavior, National Institute for Physiological Sciences, Okazaki, Aichi, Japan

*** Correspondence:**
Tsuyoshi Miyakawa, Division of Systems Medical Science, Institute for Comprehensive Medical Science, Fujita Health University, 1-98 Dengakugakubo Kutsukake-cho, Toyoake, Aichi 470-1192, Japan
miyakawa@fujita-hu.ac.jp

Yasuo Mori, Department of Synthetic Chemistry and Biological Chemistry, Graduate School of Engineering, Kyoto University, Kyoto 615-8510, Japan
mori@sbchem.kyoto-u.ac.jp

Supplementary Table 1. Comprehensive behavioral test battery of beta-anchoring and -regulatory protein (BARP) knockout (KO) mice. Age (w):age of weeks of subjects at the beginning of each test.

Tests	Age (w)
1. General health/neurological/ wire hang	11
2. Grip stength	11
3. Light/dark transition	11
4. Open field	11
5. Elevated plus maze	11
6. Hot plate	12
7. Social interaction (novel environment)	12
8. Rotarod	12
9. Sociability/social novelty	12
10. Prepulse inhibition	13
11. Porsolt forced swim	13
12. Barnes maze	14
13. T-maze	20
14. Tail suspension	36
15. Cued and contextual fear conditioning	36
16. 24 hrs home cage monitoring	42
