# Supplementary material for: Comprehensive behavioral analysis of voltage-gated calcium channel beta-anchoring and -regulatory protein knockout mice
Source: Front Behav Neurosci. 2015 Jun 16;9:141. doi: 10.3389/fnbeh.2015.00141 (PMC4468383; doi:10.3389/fnbeh.2015.00141)
Supplement: Supplementary file 2 [file Presentation1.PDF]

frontiers

Supplementary Material

Comprehensive behavioral analysis of voltage-gated calcium channel beta-anchoring and -regulatory protein knockout mice

Akito Nakao[1], Takafumi Miki[2], Hirotaka Shoji[1,3], Miyuki Nishi[4], Hiroshi Takeshima[4], Tsuyoshi Miyakawa[1,3,5]*, Yasuo Mori[2]*

Akito Nakao and Takafumi Miki contributed equally to this work.

[1]Division of Systems Medical Science, Institute for Comprehensive Medical Science, Fujita Health University, Toyoake, Aichi, Japan
[2]Department of Synthetic Chemistry and Biological Chemistry, Graduate School of Engineering, Kyoto University, Kyoto, Kyoto, Japan
[3]Japan Science and Technology Agency (JST), Core Research for Evolutional Science and Technology (CREST), Kawaguchi, Saitama, Japan
[4]Department of Biological Chemistry, Graduate School of Pharmaceutical Sciences, Kyoto University, Kyoto, Kyoto, Japan
[5]Center for Genetic Analysis of Behavior, National Institute for Physiological Sciences, Okazaki, Aichi, Japan

*** Correspondence:**
Tsuyoshi Miyakawa, Division of Systems Medical Science, Institute for Comprehensive Medical Science, Fujita Health University, 1-98 Dengakugakubo Kutsukake-cho, Toyoake, Aichi 470-1192, Japan
miyakawa@fujita-hu.ac.jp

Yasuo Mori, Department of Synthetic Chemistry and Biological Chemistry, Graduate School of Engineering, Kyoto University, Kyoto 615-8510, Japan
mori@sbchem.kyoto-u.ac.jp

Supplementary Fig. 1 Generation of beta-anchoring and -regulatory protein (BARP) knockout (KO) mice.

(**A**) Restriction enzyme maps of the wild type (WT) allele, targeting vector, expected targeted allele, and recombinated targeted allele. (**B**) Southern blot analysis of BglII-digested DNAs from BARP$^{+/+}$, BARP$^{+/-}$, and BARP$^{-/-}$ mice. The hybridization probes used are shown in (**A**). The expected sizes of the restriction fragments from BARP$^{+/+}$, BARP$^{+/-}$, and BARP$^{-/-}$ mice are 8.9, 8.9 and 16.2, and 16.2 kbp, respectively. (**C**) Northern blot analysis of BARP RNAs from BARP$^{+/+}$, BARP$^{+/-}$, and BARP$^{-/-}$ mice. (**D**) Genomic PCR analysis of DNAs from BARP$^{+/+}$, BARP$^{+/-}$, and BARP$^{-/-}$ mice. The expected sizes of the restriction fragments from BARP$^{+/+}$, BARP$^{+/-}$, and BARP$^{-/-}$ mice are 541, 541 and 1013, and 1013 bp, respectively. (**E**) Western blot analysis of BARP proteins from BARP$^{+/+}$, BARP$^{+/-}$, and BARP$^{-/-}$ mouse brains.

Supplementary Fig. 2 Distance traveled by beta-anchoring and -regulatory protein (BARP) knockout (KO) mice in the Porsolt forced swim test.

Total distance traveled on day 1 and day 2 by BARP KO and wild type (WT) mice in the Porsolt forced swim test. Data represent the mean ± SEM. The p-values indicate a genotype effect in a two-way repeated measures ANOVA.

Supplementary Fig. 3 Correlation between startle amplitude and prepulse inhibition (PPI) in beta-anchoring and -regulatory protein (BARP) knockout (KO) mice.

(**A-D**) Correlation between startle amplitude and PPI. The correlation coefficient (r) is shown in correlation Z tests.

Supplementary Fig. 4 Distance traveled by beta-anchoring and -regulatory protein (BARP) knockout (KO) mice in T-maze tests.

(**A**) Distance traveled in the training session by BARP KO and wild type (WT) mice in the T-maze spontaneous alternation test. (**B**) Distance traveled in the training session by BARP KO and WT mice in the T-maze forced alternation test. (**C**) Distance traveled by BARP KO and WT mice in the T-maze left-right discrimination test. The baited arm was changed to the opposite side from session 8 onwards. Data represent the mean ± SEM. Data were analyzed using a two-way repeated measures ANOVA. The p-values indicate a genotype effect.

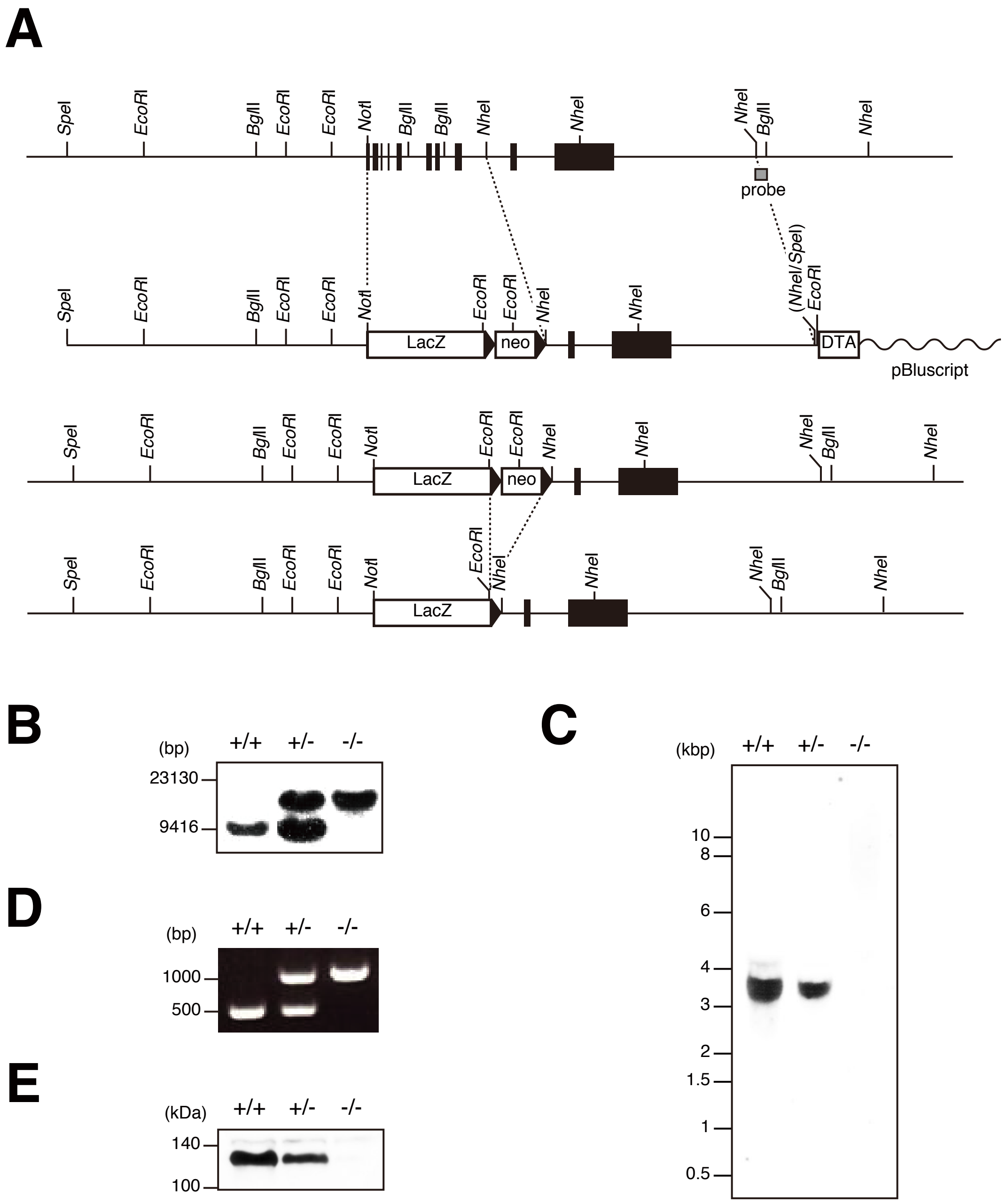

Nakao et al

Supplementary Fig. 1

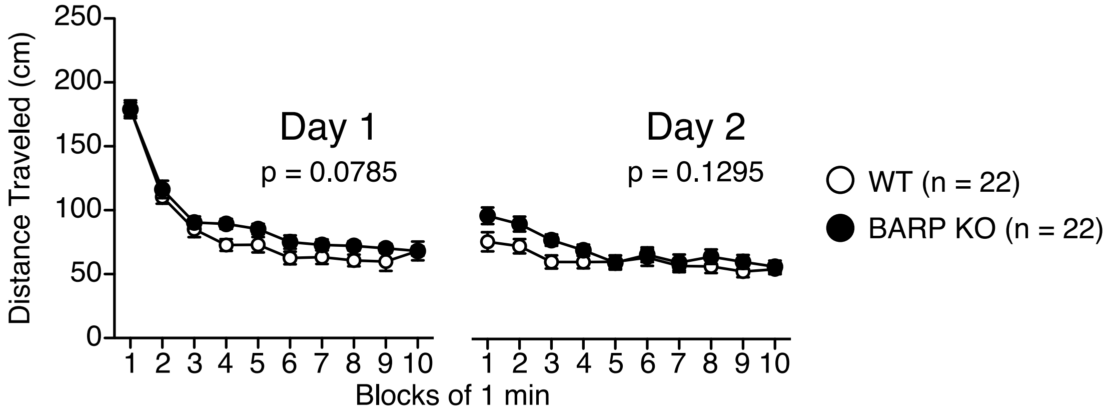

Nakao et al

Supplementary Fig. 2

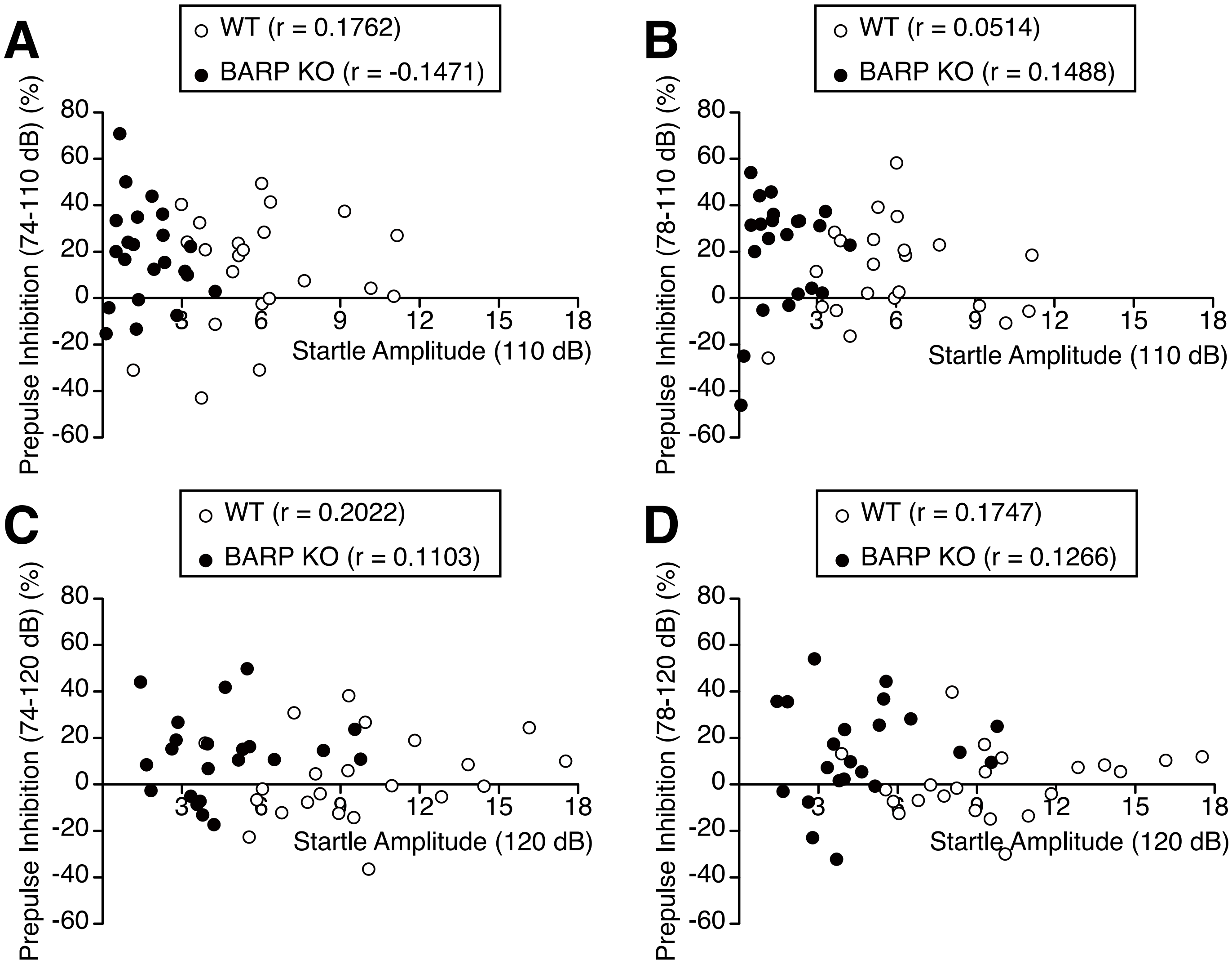

Nakao et al

Supplementary Fig. 3

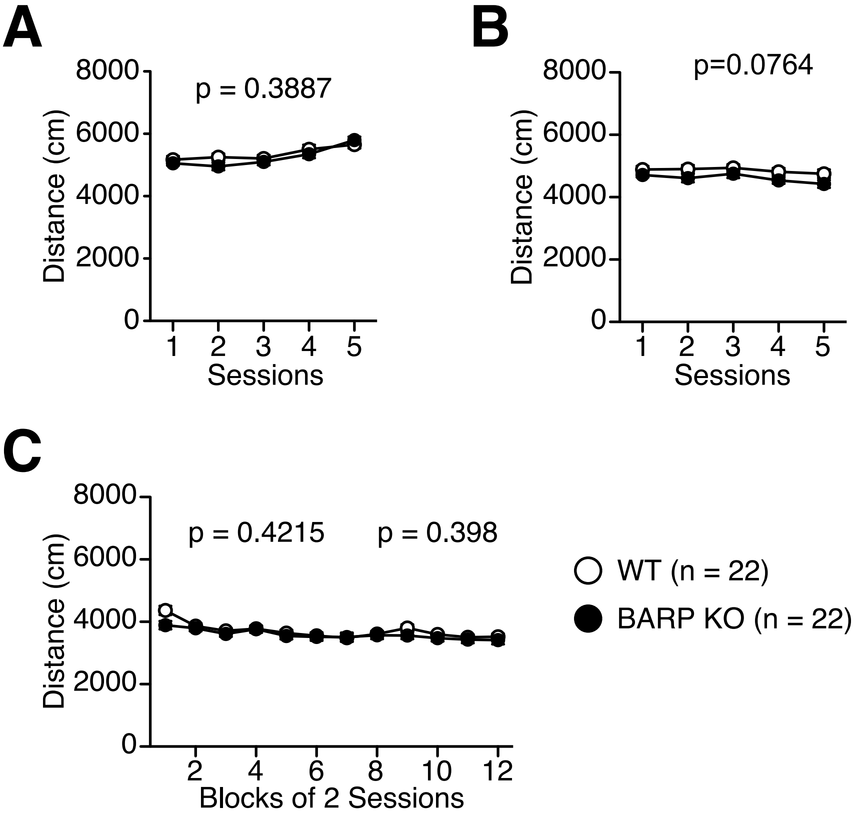

Nakao et al

Supplementary Fig. 4
